# Understanding the Implications of mHealth Technology in Collaborative Care Programs and Its Role in Supporting Postpartum Care: Qualitative Interview Study of the Baby2Home Intervention Using the Parallel Journeys Framework

**DOI:** 10.2196/70936

**Published:** 2025-08-26

**Authors:** Chryselle Rego, Joshua E Santiago, Emily S Miller, Craig F Garfield, Jacqueline Gollan, Kathleen O'Sullivan, Dinah Williams, Enid Montague, Young Seok Lee

**Affiliations:** 1 Jarvis College of Computing and Digital Media DePaul University Chicago United States; 2 The Center for Health and Social Sciences University of Chicago Chicago, IL United States; 3 Division of Maternal Fetal Medicine The Warren Alpert Medical School Brown University Providence, RI United States; 4 Feinberg School of Medicine Northwestern Medicine Chicago, IL United States; 5 Industrial Engineering University of Toronto Toronto, ON Canada

**Keywords:** mobile health, mHealth, postpartum care, collaborative care programs, mental health, Baby2Home intervention

## Abstract

**Background:**

The postpartum period represents a critical period for both birthing and nonbirthing parents due to mental health concerns and new caregiving demands. Collaborative care models aim to address these needs, but postpartum care remains fragmented, lacking continuity and holistic support. Baby2Home (B2H) is a digital intervention rooted in the collaborative care model, specifically designed to support parents through their transition into parenthood by addressing their physical, emotional, and psychosocial needs. This intervention seeks to close the gaps left by traditional care models by providing continuous, organized, and accessible support throughout the postpartum period. In our qualitative study of the B2H intervention, we reference the parallel journeys framework and use it as a part of our analysis to evaluate whether mobile health (mHealth) technology addresses the holistic needs (postpartum and psychosocial) of new parents.

**Objective:**

We aimed to assess how the B2H app supports the holistic needs of new parents and addresses care gaps identified in traditional postpartum services.

**Methods:**

We conducted semistructured interviews with 20 birthing and nonbirthing parents selected through purposive sampling based on their app use. Data were analyzed using the postpartum parallel journeys framework and inductive coding.

**Results:**

Our findings demonstrate the comprehensive impact of the B2H intervention in addressing both the physical and psychosocial needs of new parents. B2H supported postpartum care by helping parents navigate uncertainties, enhancing health care provider–parent communication, promoting self-care, and increasing parental self-efficacy. Psychosocial support included symptom identification, timely care manager assessments, coordinated treatment, and transition resources. The app also addressed care gaps by promoting inclusivity for nonbirthing parents, bridging screening and treatment, supporting real-time treatment navigation, and ensuring continuity of care.

**Conclusions:**

We demonstrate that the use of mHealth technology such as the B2H app can effectively support the multifaceted needs of new parents during their postpartum care period. By applying the parallel journeys framework, the research also identifies gaps in care that are addressed by the B2H app, presenting unique opportunities for future development and research.

## Introduction

### Background

The postpartum period is a critical phase for both birthing and nonbirthing parents as they adjust to the emotional, physical, and lifestyle demands of caring for a newborn [1,2]. Postpartum depression (PPD) is incredibly common among birthing parents, with a prevalence of 10% to 15% [3], and affects about 10% of nonbirthing parents within 3 to 6 months after childbirth [4]. Existing studies on PPD typically focus on birthing parents within the first 2 months post partum. However, a Swedish study [5] extended the time frame to 25 months after childbirth and reported depressive symptoms in 11.3% of birthing parents and 4.9% of nonbirthing parents. Beyond depression, parents face anxiety and psychological distress tied to caregiving responsibilities, which can affect marital quality and parenting behaviors [6,7].

Despite growing recognition of these challenges, postpartum care remains fragmented, often split across multiple care providers, leading to communication gaps and inconsistent support [8]. The COVID-19 pandemic further highlighted the need for scalable, patient-centered care models. In response, collaborative care programs (CCPs) have emerged as a strategic approach to address these gaps in postpartum care, aiming to provide holistic support for new parents [9]. CCPs aim to foster interdisciplinary collaboration by bringing together obstetricians, pediatricians, mental health professionals (care managers [CMs] and psychiatrists) to address the diverse needs of parents and newborns [9]. Health information technologies significantly contribute to the success of these programs by enhancing communication, coordination, and engagement between health care providers and patients. To date, these technologies have primarily included clinician-facing tools [10]. Because collaborative care is a patient-centered approach that seeks to inform and empower patients for improved self-management, incorporating a patient-facing mobile health (mHealth) tool is a logical extension of the collaborative care model (CCM) [11].

Despite the potential of mHealth technology in postpartum care and CCPs, it is crucial to note that the current body of research has primarily focused on designing technologies that support patients in managing their perinatal and postpartum mental health in day-to-day contexts [12]. The key lies in a comprehensive understanding of how mHealth interventions impact the dynamics of CCPs and the extent to which they contribute to the overall success of these programs [13].

### Embracing a Holistic Approach: Our Rationale for Developing the Baby2Home Intervention and the Need for Qualitative Inquiry

Previous studies on technology in CCPs have mainly addressed specific aspects of the postpartum experience. Some focused on mobile technologies delivering educational resources to birthing parents [14,15], while others designed interventions targeting their mental health [16-18]. Research also emphasizes that birthing parents’ support needs evolve throughout pregnancy and parenthood [19,20]. However, these studies often overlook integrated solutions that address the full postpartum care journey, leaving gaps in how parental mental health, infant care, and overall well-being intersect. In addition, most interventions prioritize birthing parents, often neglecting the significant role of nonbirthing parents in postpartum care [21]. The lack of inclusive, comprehensive programs limits support for families and hinders optimal outcomes for both caregivers and infants [22].

To address this gap, we developed Baby2Home (B2H), an mHealth intervention, based on the CCM, designed to support families holistically by meeting both physical and psychosocial postpartum needs. Unlike existing studies that focus on isolated interventions, B2H is designed to encapsulate the full spectrum of postpartum care for both birthing and nonbirthing parents.

Understanding how such an intervention fits into the broader care experience requires a qualitative approach. A qualitative study enables a deeper exploration of parents’ lived experiences, especially around how mHealth technologies influence emotional well-being, engagement with care teams, and adaptation to caregiving roles—dimensions that are not easily captured through quantitative measures.

To guide this inquiry, we applied the parallel journeys framework (PJF). Unlike most frameworks that are primarily designed for health care practitioners, the PJF is distinctive in offering a comprehensive perspective that encompasses the entire postpartum experience from the parent’s point of view. This framework uniquely integrates all facets of the postpartum journey, ensuring that the physical and psychosocial needs of parents are thoroughly addressed.

### The PJF

#### Overview

**Figure 1:**
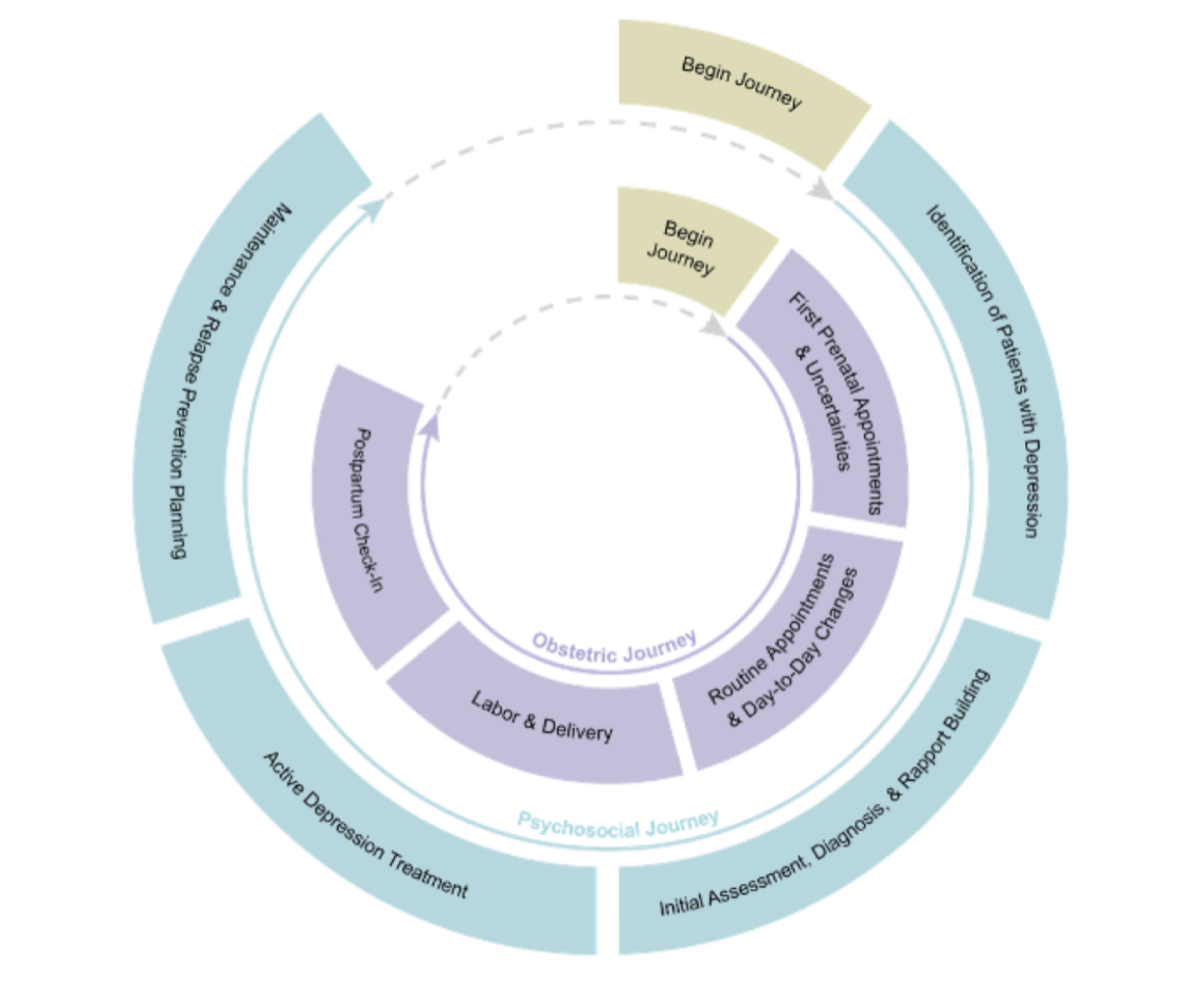
Expansion by Haldar et al [12] of the parallel journeys framework for perinatal care, representing cyclical journeys in obstetric care and psychosocial care.

The PJF, conceived by Suh et al [23], offers a lens through which the concurrent and intertwined paths of 2 distinct health care journeys can be examined, especially within collaborative care. Originally developed for collaborative cancer care, this framework sheds light on the intersecting challenges of psychosocial and cancer care journeys. Haldar et al [12] later adapted this framework for perinatal CCPs to delineate the challenges parents face at the intersection of obstetric care and psychosocial care journeys (Figure 1 [12]).

**Figure 2:**
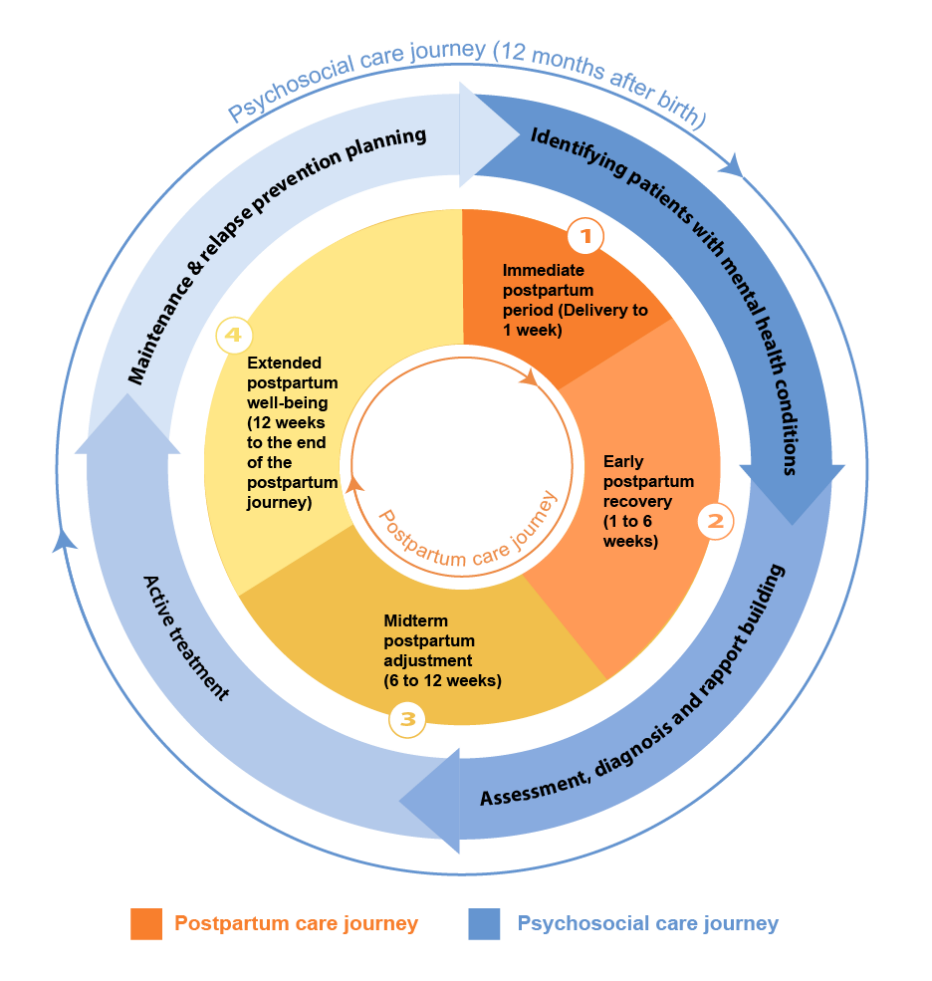
Our expansion of the parallel journeys framework highlighting the phases of postpartum care and psychosocial care.

In our study, we have modified the PJF to develop the postpartum PJF (PPJF) (Figure 2) to address the specific needs of postpartum care, which was not emphasized in the framework used by Haldar et al [12]. This adaptation was guided by a theory-driven process, drawing on the structural design by Suh et al [23] and Haldar et al [12] while integrating empirical data from our qualitative interviews. Specifically, each phase of the postpartum and psychosocial care journeys was refined through an iterative coding process informed by participant narratives and validated against existing literature. Feedback from CMs and behavioral health care providers (BHPs; therapists, counselors, and psychiatrists) in perinatal mental health and collaborative care further informed the refinement of stages to ensure clinical and experiential relevance.

The resulting PPJF captures the multifaceted progression of postpartum and psychosocial care by outlining distinct stages of transition, each marked by specific parent needs and challenges. This expanded framework offers a more holistic perspective on postpartum care and serves as the foundation for analyzing how digital interventions, specifically the B2H app, support the evolving needs of both birthing and nonbirthing parents during this critical period.

#### Phases in the Postpartum Care Journey

The PPJF delineates the postpartum care journey into 4 phases, each marked by unique challenges, needs, and transitions. These phases are derived from both the interview data and relevant literature, framing the comprehensive journey from immediate postdelivery care to long-term postpartum adjustment.

#### Immediate Postpartum Care and Transitioning Home (Delivery to 1 Week)

This phase is also known as the initial or acute period, which refers to the time following childbirth and the first few days post partum [24]. The immediate postpartum care phase is characterized by care provided to birthing parents and newborns within 24 hours after delivery. During this time, health care providers closely monitor birthing parents and infants for potential complications and offer support for breastfeeding initiation, bonding, and emotional well-being [25]. The health care team assesses uterine involution, monitors vital signs, addresses any postpartum discomfort or complications, and provides guidance on postpartum care, such as proper hygiene, pain management, and contraception options [26]. Regarding the care provided to the newborn, the postnatal check involves evaluating vital signs, conducting a physical examination, and assessing feeding patterns and weight. Health care providers offer guidance on newborn care, including feeding techniques, diapering, and safe sleep practices [26].

Transitioning home from the hospital marks a significant shift as the family assumes greater responsibility for caring for the newborn. This period involves adapting to the challenges of parenthood, establishing feeding routines, and managing the physical and emotional changes that accompany childbirth [27]. Overall, the immediate postpartum period and transition home represent a dynamic and emotionally charged time, wherein the integration of medical care, emotional support, and practical guidance sets the foundation for a healthy start to the new family’s journey.

#### Early Postpartum Recovery (2 to 6 Weeks)

The early postpartum recovery phase, also referred to as the subacute postpartum period, spans the first 6 weeks after childbirth and encompasses the initial weeks of physical and emotional adjustment for new birthing parents [24]. Physically, this phase involves the body’s gradual recovery from the demands of childbirth. Birthing parents may experience uterine contractions, vaginal healing, and hormonal fluctuations during this time [24]. Emotionally, the postpartum period can bring about a range of feelings, from joy to potential mood fluctuations. In this context, the preparation for the first postpartum visit gains significance.

The first postpartum visit marks an important phase in the continuum of care for both the birthing parent and the newborn. Typically occurring within the first 2 to 4 weeks postpartum, this visit acts as a pivotal touchpoint for health care providers to assess the physical and emotional well-being of the birthing parent [24]. During this phase, the obstetric care provider conducts a physical examination and administers mental health symptom screening to assess health concerns that might have emerged during delivery [10]. The physical examination includes a pelvic examination and checking the birthing parent’s blood pressure, weight, and temperature. In case of a vaginal tear or c-section incision, the obstetrician checks to ensure that the stitches are healing [28]. A breast examination is also conducted to assess for engorgement or any changes that can occur with breastfeeding and pumping [29]. As part of the mental assessment, the obstetric care provider uses a paper-based screening tool to assess the risk for PPD. By addressing the physical and emotional aspects of postpartum recovery, the first postnatal visit lays the foundation for ongoing support, promoting the well-being of both birthing parents and the infant.

#### Midterm Postpartum Adjustment (6 to 12 Weeks)

The midterm postpartum phase, spanning from 6 to 12 weeks after childbirth, is a pivotal period for new parents as they further adapt to the responsibilities of parenthood. This phase is characterized by continued physical recovery from childbirth and deeper emotional adjustments as parents settle into their new roles [30]. During this time, the initial intensity of postpartum recovery begins to wane, and parents may start to feel more physically comfortable [26]. However, this period also brings its own set of challenges and adjustments. Physical recovery may still be underway, with some women experiencing lingering postpartum symptoms, such as pelvic floor issues, hormonal changes, and fatigue [31]. Emotionally, this period can be intense, with new parents grappling with feelings of anxiety, and in some cases, signs of postpartum mood or anxiety disorders [3]. While parents may start to feel more confident in their caregiving abilities, the reality of ongoing sleep deprivation, hormonal adjustments, and the challenges of balancing personal and professional responsibilities can contribute to feelings of fatigue.

As parents approach the end of the midterm postpartum phase, a comprehensive postpartum checkup is recommended [32]. This comprehensive evaluation aims to provide a detailed review of the birthing parent’s physical, emotional, and psychosocial health, covering critical areas, such as emotional well-being, care and feeding of the infant, sexual health, contraception, and planning for future pregnancies, as well as management of sleep, fatigue, physical recovery, chronic conditions, and overall health maintenance. For most patients, the comprehensive postpartum appointment is their final touchpoint with their obstetric care provider.

#### Extended Postpartum Well-Being and Long-Term Adjustment: (3 Months and Beyond)

The extended postpartum well-being phase, also known as the delayed postpartum period, emphasizes a comprehensive and ongoing approach to health for both the birthing parent and infant [24]. As this phase unfolds, the needs of parents evolve while they adapt to more settled routines and face the sustained elements of postpartum care.

Birthing parents experience a multifaceted mix of physical and emotional adjustments during this stage, especially as they transition back to work or adjust to their partners doing so. Physical recovery from childbirth may still be underway, with women navigating changes in skin and hair, hormonal fluctuations that can impact mood and physical well-being, the gradual return of menstruation, and efforts toward weight management [33]. Changes during this phase are extremely gradual. Although change is subtle during this phase, it is important to remember that a birthing parent’s body is not fully restored to its prepregnant state until about 6 months after delivery.

Furthermore, the challenges of balancing professional responsibilities with the demands of parenthood and the added stress of securing suitable daycare arrangements can further contribute to elevated stress levels. This period marks a critical juncture where ongoing monitoring of mental health becomes essential as parents navigate through the complexities of returning to work and maintaining the well-being of their expanding family.

#### Phases in the Psychosocial Care Journey

For new parents, psychosocial well-being encompasses a broad range of factors that contribute to mental, emotional, and social health. Here is a detailed overview of how the psychosocial care journey unfolds within the context of postpartum care.

#### Identification of Patients With Mental Health Conditions

This phase acknowledges that while it is common for new parents to experience a broad range of emotions, such as periods of sadness, known colloquially as “baby blues,” there’s a crucial distinction to be made when these feelings persist or intensify, potentially indicating PPD, a more serious condition [32]. Typically occurring at the 2- to 4-week mark, this phase represents a birthing parent’s first postpartum encounter with their obstetrician. During this initial postpartum visit, the obstetric care provider uses standardized paper-based measures, such as the Edinburgh Postnatal Depression Scale [34] or the Patient Health Questionnaire [35], to screen patients for symptoms of PPD. If a parent exhibits symptoms or has a known history of mental health issues, the health care provider introduces CCM as a valuable resource. The health care provider discusses the potential benefits of connecting with CCP and gauges the patient’s interest in a referral. However, it is important to note that most perinatal care offices do not have a CCM available on-site and often refer parents to external mental health support.

The significance of this stage lies in its emphasis on timely intervention and the establishment of a supportive care environment that prioritizes mental health. Early identification allows for the timely implementation of support systems and interventions helps promote the overall well-being of both the parent and the newborn [36].

#### Initial Psychosocial Assessment, Diagnosis, and Rapport Building

Following the detection of symptoms of PPD, birthing parents who screen positive and have a clinical assessment congruent with PPD are referred to a BHP by their obstetric care providers. This process is designed to gather detailed information about the patient’s symptom severity. The BHP conducts a thorough psychosocial assessment using standardized tools to establish baseline symptom severity (eg, 9-item Patient Health Questionnaire [PHQ-9] and 7-item Generalized Anxiety Disorder [GAD-7]) [35,37]. In addition to these quantitative measures, the BHP also dives deeper into the patient’s personal history, gathering information on a range of factors that could influence their mental health. This includes exploring the patient’s family and medical history, which can reveal genetic predispositions or health conditions that may impact mental well-being [38]. Psychiatric and substance use history is also reviewed, as previous or current issues can significantly affect treatment planning and outcomes. In addition, understanding the patient’s financial background and social support systems is crucial, as financial stressors and isolation can exacerbate mental health problems [38]. However, it is important to note that despite the critical importance of the referrals provided by the obstetrician, recent data suggest that fewer than 1 in 10 people referred to BHP actually engage with behavioral health services. While an obstetrician may refer a patient to a BHP, this transition often does not occur as intended. Patients frequently face challenges navigating the process, such as identifying the right contact, scheduling an appointment, finding health care providers who accept their insurance, and securing timely appointments close to home. This represents a significant barrier and highlights a substantial gap in the current health care system.

This phase sets the stage for a personalized treatment approach, which is essential for successful outcomes in mental health care. The assessment conducted by the BHP ensures that the subsequent steps in the treatment process are based on a solid understanding of the patient’s psychosocial context, facilitating targeted interventions and individualized care plans.

#### Active Depression Treatment

The active treatment phase for PPD involves a targeted and evidence-based approach [23]. The treatment plan administered during this phase is highly individualized, considering the patient’s unique circumstances, preferences, and needs to ensure personalized care [36]. In most clinical settings, when a birthing parent screens positive for depression during their 6-week postpartum visit, the obstetrician prescribes medication. If the patient is receiving mental health care, such as therapy, the clinician will monitor their progress and intervene as necessary. In some instances, insurance providers employ care coordinators who contact postpartum parents at some point after birth to assess for PPD and connect them to appropriate support services.

In the context of PPD, active treatment may involve various therapeutic approaches, including supportive counseling, psychotherapy, and medication management. For parents experiencing mild to moderate depression, psychological interventions, such as cognitive behavioral therapy or interpersonal therapy, are recommended [33]. These therapies aim to enhance coping strategies, address interpersonal issues, and modify negative thought patterns that contribute to depressive symptoms. For those with moderate to severe depression, a combination of psychotherapy and antidepressant medications may be advised [39]. Medications are chosen with care, considering the patient’s breastfeeding status and personal preferences to ensure both safety and effectiveness.

The goal of the active depression treatment phase is not only to reduce symptoms of depression but also to empower parents to manage mental health effectively. This process is dynamic and responsive, characterized by a collaborative partnership between the patient and their health care team. Such an approach ensures that treatment aligns with the patient’s recovery goals and adapts to their changing needs, ultimately paving the way for a successful recovery from PPD.

#### Maintenance and Relapse Prevention Planning

The final phase of the psychosocial care journey plays a crucial role in sustaining the well-being of new parents after active treatment has concluded [39]. This phase is designed to equip patients with the resources needed to maintain their mental health and prevent the recurrence of depressive episodes. It acknowledges the reality that the journey through PPD is not solely about overcoming the immediate symptoms but also about building a resilient foundation for long-term mental health [40].

In this phase, BHPs work closely with parents to develop a comprehensive maintenance plan (eg, identifying early warning signs or establishing effective coping strategies) that addresses each individual’s unique needs and circumstances [23]. Ideally, postpartum care should extend up to 1 year after birth, as postpartum-related mental health symptoms can often persist throughout this period [12]. However, most clinical settings lack CCMs, with obstetric care typically concluding within 6 to 8 weeks post partum, leaving a gap in ongoing support. Toward the end of obstetric care, BHPs proactively engage with individuals to discuss the end point of their program involvement. In cases where additional support is needed, BHPs provide external referrals, ensuring that patients can access longer-term therapy or treatment in locations convenient to them [39]. BHPs also encourage parents to engage in self-care practices, such as regular physical activity, healthy eating, and mindfulness exercises, which can contribute to overall well-being and resilience.

In conclusion, the fourth phase helps secure the progress made during the active treatment of PPD and lays the groundwork for sustained mental health. It represents a transition from intensive care to a more self-directed approach, with continued professional support tailored to prevent relapses and promote long-term recovery. Although perinatal care concludes at the 1-year postpartum mark, a patient’s mental health treatment may continue beyond this end point outside the postpartum context.

### The B2H Intervention

B2H is an mHealth intervention built on the CCM designed to support families during their transition to parenthood. Upon being allocated to the intervention arm, families are introduced to the B2H services through a mandatory download and use of the B2H smartphone app, compatible with both iOS and Android platforms. The app incorporates features designed to support both parent wellness and infant care. These features cover essential postnatal care practices, including, but not limited to, tracking mechanisms for feeding, sleeping patterns, growth milestones, diaper changes, and vaccination schedules. Moreover, the app engages parents with educational content on infant developmental milestones, conducts monthly mental health assessments, and supports adherence to preventive health care schedules for both the parents and their newborns.

The B2H intervention is structured to offer a holistic suite of services aimed at reinforcing the well-being of both parents and infants (Multimedia Appendix 1 [41]). If a parent screens positive for moderate stress (eg, a Perceived Stress Scale [PSS] score of 14-26), the CM reaches out to the parent via chat to assess the parent’s stress situation and recommends stress management resources, such as the Stress Management and Resiliency Training module, that teaches self-care practices to manage daily stress and regain a sense of emotional control. If severe stress is identified (eg, PSS≥27), the CM contacts the parent directly to assess the source of stress and provides psychoeducation on stress management techniques.

On the other hand, if a parent screens positive for depression or anxiety (PHQ-9≥10; GAD-7≥10), the CM will contact the patient to facilitate a clinical assessment and initial treatment planning. For individuals who desire psychotherapy, brief behavioral care (8 sessions of behavioral activation) will be initiated, led by the CM via phone. For individuals who desire pharmacotherapy, the CM will refer the participant to a primary care physician or a psychiatrist, with the decision regarding the type of referral informed by the clinical context.

If the assessment scores indicate a level of concern, the CM consults with a clinical psychologist to discuss recommendations for supporting the patient in accessing the appropriate care. This may involve assisting the parents in developing a safety plan and connecting them with the necessary behavioral health services. In more severe cases, this could include contacting the OB or an emergency contact if needed. Once the recommendations are agreed upon, the CM follows up with the parent to ensure that the proposed plan is acceptable and discusses next steps. Through this personalized and supportive approach, B2H aims to enhance the user experience, providing guidance and resources to ensure the parent receives the care they need throughout their postpartum journey.

## Methods

### Study Design

This research is carried out in parallel with a National Institutes of Health–funded (grant #1R01HD105499-01) randomized controlled trial (RCT; NCT05595486) [42] that attempts to investigate the efficacy of mHealth apps (in particular B2H) on preventive health care use for the family unit and patient-reported outcomes trajectories with a focus on mental health. As part of the RCT, 642 diverse families have been randomly assigned to either usual care or the innovative B2H intervention to evaluate the efficacy of B2H health services use and patient-reported outcomes.

**Figure 3:**
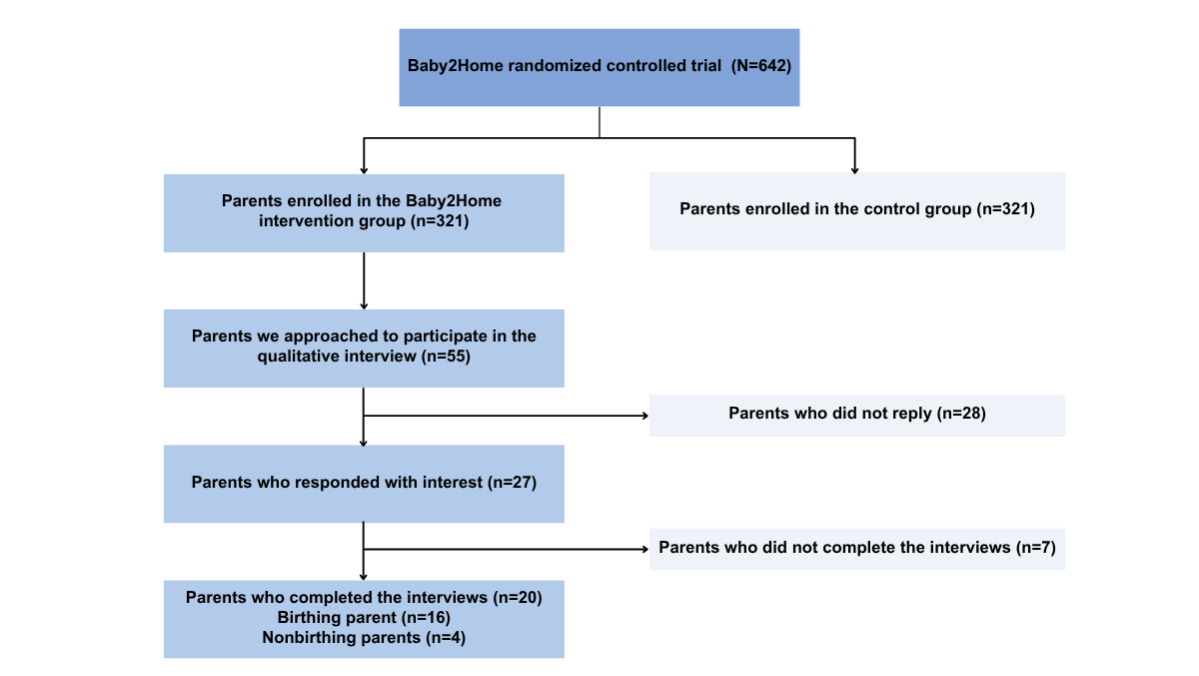
Flow diagram of the qualitative study participants in the intervention group.

This paper, while part of a larger study, specifically focuses on the intervention group. In this group, families (n=321) received the specialized services offered by the B2H app (see Figure 3). In this qualitative study, we aimed to explore the engagement and experiences of both birthing and nonbirthing parents within the intervention group as they interact with the B2H app.

Importantly, this qualitative component offers critical context to the broader findings of the RCT by helping to explain potential mechanisms behind intervention efficacy. While the RCT will evaluate user engagement quantitatively through backend app analytics and assess perceived app efficacy using the User Version of the Mobile Application Rating Scale, this study provides insight into the “why” behind the use patterns, such as the multifaceted needs of new parents during the postpartum period and how these evolving needs influence app engagement. These qualitative findings help contextualize quantitative trends in use, satisfaction, and outcomes, offering actionable design recommendations to enhance intervention effectiveness and inform future iterations of the B2H app.

### Participant Recruitment and Interviews

A purposive sampling technique was used to select a diverse group of participants from the B2H intervention arm, specifically targeting parents in the 4- to 8-month postpartum period with varying levels of app engagement, categorized based on cumulative time spent on the app. App use was calculated by taking the average of the total number of sessions (number of times the app was opened) and dividing it by the number of participants in the intervention group. Participants were then classified into 2 groups: those with fewer than 175 total sessions were categorized as low use and those with more than 175 sessions as high use (Multimedia Appendix 2). However, it is essential to note that some individuals classified as low app use users demonstrated substantial engagement in specific features of the app, including baby tracking, chat functionality, content consumption, and participation in mental health assessments.

Semistructured interviews facilitated the collection of detailed narratives concerning the participants’ use of the B2H app and its impact on their postpartum experience. These interviews, lasting between 45 and 60 minutes, were conducted by the first author via videoconferencing software (Zoom Communications Inc). The semistructured interview schedule was developed by the research team and informed by the objectives of the larger RCT, as well as existing literature on postpartum care, digital health engagement, and CCMs. The interview questions (Multimedia Appendix 3) sought to uncover patterns of app use; impacts on health care accessibility; engagement with the app’s specific features (eg, baby tracking, mental health assessments, and educational resources); perceived impacts on postpartum experiences; and areas for improvement. To ensure clarity and relevance, the guide was pilot-tested with 2 participants and refined based on team discussion and feedback from early interviews.

The recruitment process involved systematically disseminating email invitations and providing potential participants with comprehensive details about the study, including its objectives, procedures, and potential benefits. A notable proportion of parents who were initially contacted did not respond, as many of them had recently returned to work and reported difficulty balancing work responsibilities with the demands of caring for a newborn, making it challenging to find time for an interview. In some cases, parents expressed interest but ultimately could not commit due to time constraints. To mitigate this, we followed up with additional emails and initiated phone calls to encourage participation. This iterative recruitment strategy aimed to maximize participant involvement and ensure the diversity of perspectives within the study cohort.

### Sample Size and Data Saturation

A subset of 20 parents, exhibiting both low and high app use based on the cumulative time spent on the B2H app in the 4- to 8-month postpartum period, consented to participate in the semistructured qualitative interviews. The participants selected for qualitative interviews exhibited a diverse range of demographic characteristics. The demographic composition included 15% (3/20) of the participants identifying as Hispanic or Latinx, 10% (2/20) as Black or African American, and 75% (15/20) as European or White. Notably, the study cohort reported various postpartum challenges, such as neonatal intensive care unit admissions, PPD, preeclampsia, a fourth-degree tear during childbirth, and previous complications such as miscarriage. A subset of participants (7/20, 35%) had a history of managing one or more pre-existing mental health conditions, including depression, anxiety, bipolar disorder, and attention deficit hyperactivity disorder, before giving birth. These diverse characteristics enrich the qualitative exploration of the B2H app’s impact on postpartum care experiences within this unique participant cohort.

Data saturation was reached during the interview process. This was determined when no new themes or patterns emerged from the data, and additional interviews yielded redundancy in responses. The research team monitored for saturation iteratively, and once the analysis of the last few transcripts confirmed that further interviews were unlikely to generate new insights, data collection was concluded.

### Data Analysis

We adopted a mixed methodology for our data analysis. The PPJF was used as a guiding lens to organize and interpret our analyses. The phases of the PPJF were first created, after which we adopted an inductive, open-coding methodology for our data analysis. A total of 21 codes were generated, and the identified themes were subsequently organized into different phases of postpartum care and psychosocial care. This process involved multiple team members to ensure a comprehensive and reliable coding framework. The research team applied the coding framework to all transcripts using qualitative data analysis software (ATLAS.ti; Lumivero, LLC). At least 2 team members coded each transcript to ensure reliability and consistency in coding. Any discrepancies or disagreements in coding were resolved through discussion and consensus among team members.

Following the completion of the data analysis, the PPJF was used to organize and interpret the results. Gaps identified in each phase were noted and informed by the qualitative codes generated during the analysis. We also made modifications to the framework proposed by Haldar et al [12] to better align it with the intricacies of the postpartum experience, offering a deeper, more nuanced perspective on the participants’ experiences and the role of mHealth technology in navigating the complexities of collaborative care settings.

### Rigor and Trustworthiness

To strengthen the trustworthiness of our qualitative findings, we incorporated several strategies throughout the data collection and analysis process by adhering to the guidelines proposed by Lincoln and Guba, as reported in the study by Thomas [43]. Credibility was ensured through triangulation, involving multiple coders who independently analyzed transcripts and resolved discrepancies through discussion and consensus. This meticulous approach ensured that various perspectives were considered, enhancing the reliability and validity of the data analysis. Dependability was enhanced by maintaining a detailed audit trail of coding decisions, analytic memos, and theme development. Confirmability was supported through regular debriefings among the research team to reflect on biases and maintain analytic neutrality. Finally, transferability was addressed by providing rich descriptions of the study’s themes and supporting them with direct quotes, all of which helped to support the validity of our analysis and contribute to the study’s trustworthiness.

### Ethical Considerations

The research protocol adhered to the ethical standards set forth by the institutional review board of DePaul University (research protocol #RB-2024-1203), ensuring compliance with the established guidelines. Informed consent was obtained from all study participants prior to data collection, and participation was voluntary. To safeguard participant privacy, all identifying information was removed, and data were deidentified before analysis. To acknowledge participants’ time and effort, compensation was provided in the form of $50 for participation in the interviews, along with additional payments for completion of assessments: $20 for the baseline, 1-month, 2-month, 4-month, and 6-month assessments, and $30 for the 12-month assessment.

## Results

**Figure 4:**
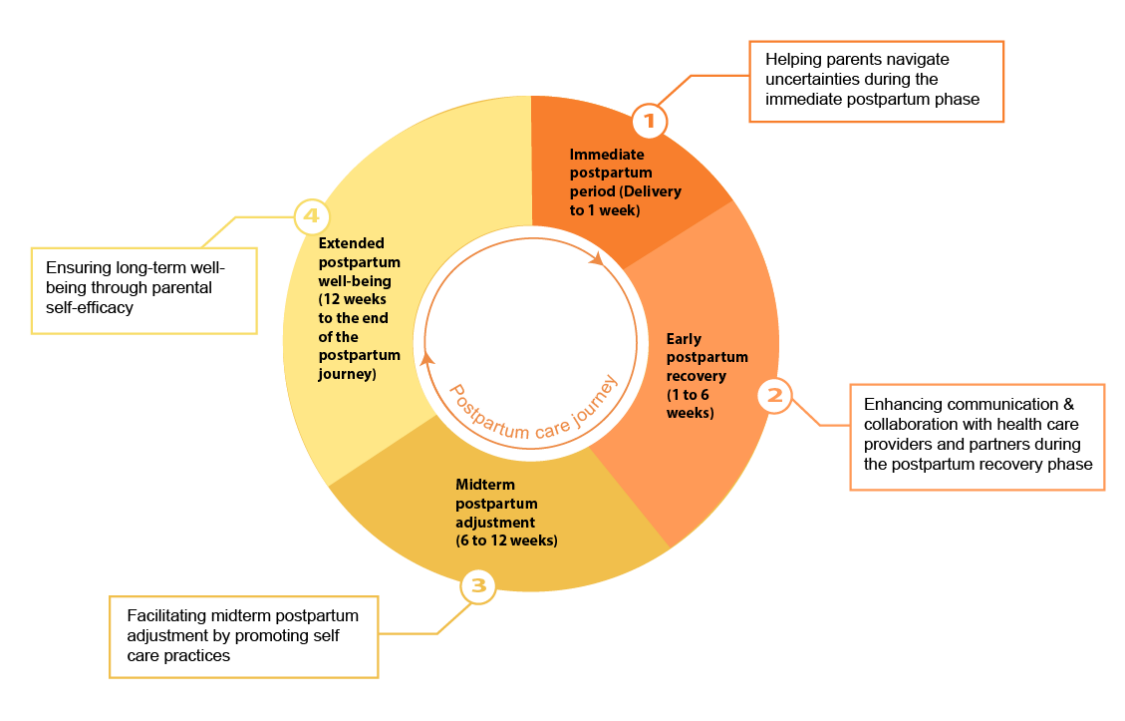
Parents reported outcomes indicating app support during the postpartum care journey.

**Figure 5:**
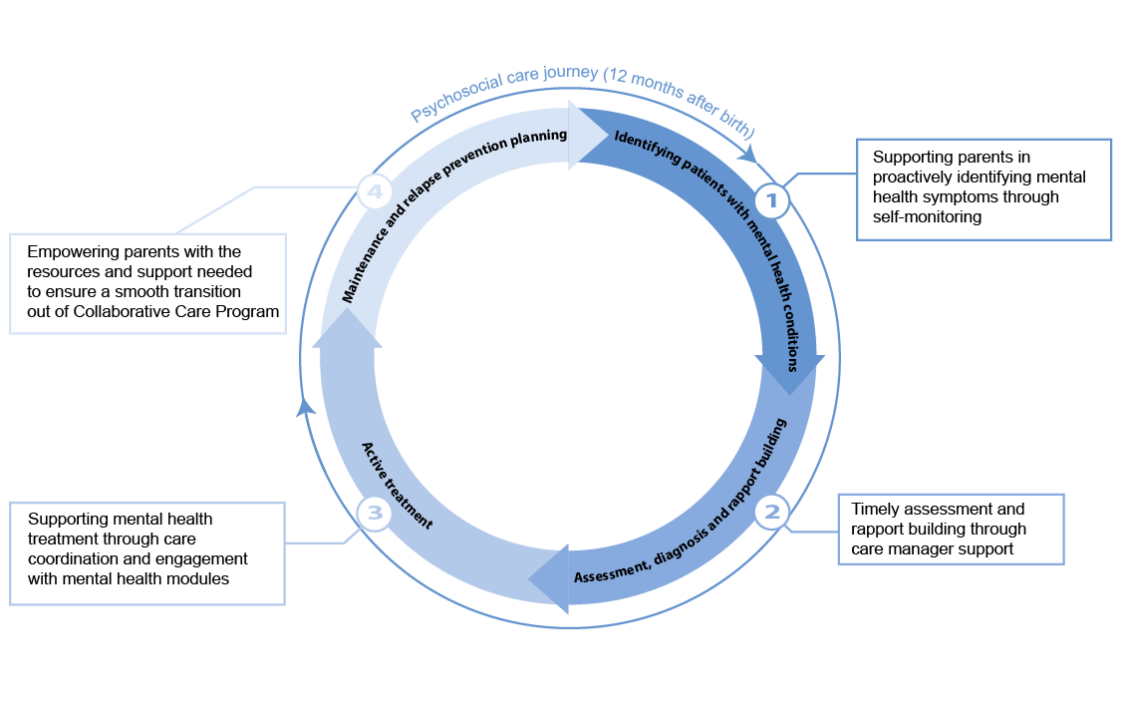
Parents reported outcomes indicating app support during the psychosocial care journey.

To provide a comprehensive analysis of the B2H app’s impact, our findings are organized into 2 main areas: postpartum care outcomes (see Figure 4) and psychosocial care outcomes (see Figure 5). This structure not only highlights the multifaceted role of the app in addressing the diverse needs of new parents but also underscores the potential of mHealth technology to bridge gaps in traditional care practices.

### Parent-Reported Outcomes Indicating App Support During the Postpartum Care Journey

#### Theme 1: Helping Parents Navigate Uncertainties During the Immediate Postpartum Phase

Parents in the intervention group reported that the educational articles and the baby tracking features in the B2H app played an essential role in guiding new parents through the multifaceted challenges of this period. Birthing parents, in particular, found solace in the app’s extensive library of educational content aimed at easing the transition after childbirth.

One parent explained as follows:

5 months into postpartum, I definitely feel better mostly, because I have had a lot of time to just read articles on self-care and de-stressing. As a new parent you tend to prepare a lot for everything surrounding baby care. Everyone tells you that you need this or that but no one really tells you how you’re gonna be. I don’t think you could really prepare for how you will be doing mentally after giving birth. B2H has been helpful in assisting me navigate those uncharted waters in those earlier weeks.

P7; birthing parent; low app use

Other parents reported that the targeted educational content provided within the app on postpartum discomfort and recovery offers essential support for new parents during the critical initial weeks following childbirth. These resources were especially valuable for parents who were uncertain about the postpartum health conditions they could encounter.

One parent reflected as follows:

I read the article on B2H about the signs and symptoms of preeclampsia and how you can get it up to six weeks postpartum. And guess what, I had six of the seven symptoms that were listed in that article. This prompted me to call my physician and I went in for an appointment that day, ended up being admitted and was on a magnesium drip for 24 hours. It was pure hell! But that article really helped me solidify that yes, I am definitely not going crazy and this isn’t just postpartum psychosis, which I was feeling at that point. I was valid in my reasoning and needed to ask for help.

P10; birthing parent; low app use

In the realm of newborn care, parents reported a heightened reliance on the B2H app’s baby tracking feature within the app during the initial weeks following childbirth. This heightened engagement with the app can be attributed to the myriad changes and challenges associated with the transition to parenthood.

One participant shared their experience, stating the following:

Our daughter was born a little underweight than most babies, and the immediate concern for us was feeding. Just because it was difficult in the hospital, to get her to latch. Being new parents, the scariest thing in the world is the thought that maybe you’re doing something that might not be leading to the right type of nourishment. To be able to get some answers on the app on what’s normal as far as breast milk production, which is what the article covered, was very useful. The app was helpful to keep track of the feedings and have a timestamp of when you last fed so that you can make sure that you’re staying on a feeding schedule.

P2; nonbirthing parent; high app use

Another parent reflected on their breastfeeding challenges and the role that B2H played in supporting them:

As a new parent, you tend to question every decision made and often underestimate mom guilt. Breastfeeding was not the easiest thing in the world at the start. The article from September 28th, regarding breast milk regulation, which I was able to discuss with my wife, was very useful. To be able to get some answers on what’s normal as far as breast milk production which is what the article covered was really helpful. It gave me the confidence to manage breastfeeding on my own terms without going through the hassle of finding and contacting a lactation consultant.

P20; birthing parent; high app use

#### Theme 2: Enhancing Communication and Collaboration With Health Care Providers and Partners During the Postpartum Recovery Phase

The early postpartum recovery phase spans the first 6 weeks after childbirth, encompassing the initial weeks of physical and emotional adjustment for new parents. During this phase, most participants reported an increased engagement and reliance on the baby tracking feature and the library of evidence-based resources on postpartum care and newborn health. According to the feedback provided by the parents, the educational articles and the tracking features served as a key preparatory step for their upcoming postpartum visit and well-child checkup.

In the days leading up to the postpartum visits and the well-child visit, the parents described how the app acted as a valuable preparatory tool, ensuring that they felt confident in discussing their own progress with the obstetricians during the postpartum visit and their newborn’s progress during the well-child visit.

One parent reported the following:

I do love that there’s an option for tracking and added notation. We were able to have a well laid out list or any notes that we had about certain things. This made it easy for me to give detailed responses to the obstetrician’s questions about breastfeeding, latching, or ask the pediatrician any specific questions about our baby.

P1; birthing parent; low app use

Another parent explained as follows:

I struggled for the longest to remember every single thing associated with baby care such as time spent breastfeeding, diapers changed etc. Having to remember all these small details got me stressed out even more. The articles on the app helped shed light on our baby’s nutrition. I am now more aware of my baby milestones and what to expect specifically in terms of growth. I see a dip in his weight and the app helps alert me and allows me to reach out to the pediatrician and get the help needed. This helps reduce any anxieties or uncertainties. Just seeing my baby’s weight and height increase on the growth chart within the app gives me this sense of relief because at one point my baby did lose weight which was concerning.

P6; birthing parent; high app use

In addition to improving communication with health care providers, birthing parents also mentioned how the app enhanced communication and collaboration with their partners during the early postpartum recovery phase. Birthing parents recovering from the physical demands of childbirth emphasized their reliance on the baby tracking feature as a means of distributing caregiving responsibilities effectively. This feature allowed them to share the workload and ensure that both parents were equally involved in the care of their newborn, fostering a more balanced and supportive environment during this critical period.

One participant recounted as follows:

B2H really helps take out all the guesswork in parenting. Before having our baby, my husband and I were actually looking for apps that we could both use simultaneously to input the baby’s information. There were other apps that we downloaded but I could only see my entries and my husband couldn’t see how many times I had fed the baby or how many times the diapers had been changed or when she last slept. With the B2H app we could see each other’s entries which allowed us to be on the same page. We would take turns feeding and used the app as a reference tool to track when the baby was last fed and how much the baby had eaten. I was able to look at the app and say that the baby ate half an hour ago, and my partner would get the bottle prepared when she was ready to eat again within the next 30 minutes.

P9; birthing parent; high app use

#### Theme 3: Facilitating Midterm Postpartum Adjustment by Promoting Self-Care Practices

Parents progressing through the midterm postpartum period reported a noticeable shift in their app use behavior, with a growing reliance on educational content within the B2H app. According to their feedback, this evolution reflects their increasing comfort and confidence in addressing their baby’s basic needs, prompting a broader exploration of topics, such as self-care, postpartum well-being, hormonal adjustments, and fatigue management strategies.

One parent reported the following:

The baby tracking feature was useful during those initial checkups, allowing me to answer very specific questions about the frequency of feeding and diaper changes. However, in subsequent checkups, I noticed a decrease in the request for such information. Initially, my primary concern centered around my baby’s weight, leading to extensive use of the baby tracking feature. However, during subsequent visits, the pediatrician gave me some confidence by saying that as long as my baby stays on her weight curve, there is no reason to be concerned and we can now start feeding on demand. This allowed me to move away from being metric centered and take some time and prioritize my own wellbeing.

P8; birthing parent; low app use

As parents become more adept in recognizing their baby’s cues and patterns, their transition from heavy use of tracking features to increased focus on educational content signifies a positive evolution in their parenting journey. This shift in app use reflects a natural progression from the intensive tracking required in the immediate postpartum period to a more holistic approach that encompasses a broader understanding of self-care and infant care. Birthing parents, in particular, mentioned how the app addressed this need by providing them with a rich repository of evidence-based information and resources, including articles and educational resources that guide physical recovery, hormonal balance, and improved rest and energy.

One parent shared the following:

As new parents, your needs are constantly changing almost every other month. In my case, the need for documentation and tracking was a lot higher during those earlier weeks, gradually transitioning as time passed. I really enjoyed the articles on postpartum recovery. I thought that they were incredibly helpful and gave me some useful tips to manage my fatigue and adjust to the hormonal changes. This made me feel more in control of my recovery and less overwhelmed.

P12; birthing parent; high app use

Another parent emphasized how the app supported their self-care routine:

The educational content surrounding self care was great. After you have a baby you forget about yourself. Having that content is a nice reminder to check in on yourself. I feel like it gave me a second to breathe and understand what I should be doing. It gave me a different perspective and resources on things to do. I needed to hear that from an outside source, especially when I was having a really bad day. The content made me feel like it was okay to leave my baby in the crib if I needed to. It gave me different avenues to alleviate my stress and know that this stress is just temporary and it will pass.

P10; birthing parent; low app use

As parents approach the end of the midterm postpartum phase, a comprehensive postpartum checkup is recommended. This evaluation aims to provide a detailed review of the birthing parent’s physical, emotional, and psychosocial health. Birthing parents who attended their comprehensive postpartum checkup reported ways in which the app helped them prepare for this critical consultation:

The app allows for the tracking of physical symptoms, emotional states, and key milestones, all of which are useful when talking to my provider. B2H does a good job providing parents with checklists and question prompts which in my case helped me articulate concerns and queries regarding self care and coping with any lingering postpartum challenges. In a way it helped me have a thorough and productive checkup.

P16; birthing parent; high app use

On the other hand, parents who failed to attend their comprehensive postpartum visit mentioned how the educational articles within the app effectively covered any critical areas they might have missed during the comprehensive visit.

One parent remarked as follows:

I attended my first postpartum visit and was scheduled for a follow up visit which I ended up skipping as I felt fine for the most part. That being said I don’t think I missed out on much as the app’s articles pretty much made up for it. They had detailed information on everything from mood and emotional well-being to contraception and planning for future pregnancies. It pretty much covered everything I needed to know about my own recovery.

P14; birthing parent; high app use

#### Theme 4: Ensuring Long-Term Well-Being Through Parental Self-Efficacy

Another significant theme that emerged from the qualitative interviews was the pivotal role that the B2H app played in facilitating long-term adjustment and well-being for new parents as they transitioned from intensive postpartum care to more self-directed management of their physical and mental health. Parents noted that the timely articles and targeted educational content within the app offered them a comprehensive suite of tools designed to enhance their self-efficacy.

In terms of maternal care, birthing parents reported that the weekly articles encouraged them to engage in regular physical activity, healthy eating, and mindfulness exercises, all tailored to fit into their busy schedules. Parents also reported that the app provided them with educational content focused on identifying triggers, managing stressors, enhancing self-efficacy, and empowering parents with the knowledge and skills to navigate future challenges independently.

One parent expressed the following:

The articles helped me think and reflect on what I am feeling. They help normalize aspects of my mental health. For instance the article shared with me yesterday by my care manager on grounding and de-stressing techniques reminded me to take some time for self-reflection whenever I feel anxious or stressed. It’s helpful to have these resources to refer back to and allows me to practice different ways of coping with stress such as breathing and meditation exercises.

P6; birthing parent; high app use

Parents in the extended postpartum phase who were navigating the challenges of returning to work mentioned how the personalized content that was tailored to their baby’s age and parenting journey addressed their evolving needs during the postpartum period.

A birthing parent noted as follows:

While many online resources cover postpartum care, few actually do a good job preparing you for transitioning back to work and not having the rest you previously did. Because you go from work to having to take care of your child so it’s like work and then a different type of work that is full of warmth and fuzzies, but can be exhausting. I remembered coming across some articles on B2H which in a way helped me mentally prepare for this shift and helped me realize that it would be helpful to track my stress levels during this period. Both my wife’s and my anxiety levels were slightly elevated compared to our General Anxiety Disorder scores before we resumed work. I checked in with my care manager around this time and she assisted me with daycare options and told me that she can help me find a therapist in my neighborhood to ensure that my new routine did not come in the way of my personal wellbeing.

P17; birthing parent; high app use

Another nonbirthing parent mentioned the following:

The educational content is laid out where it kind of feels like it’s growing with our daughter. The content is targeted towards us during the six to twelve week period which is huge. If this continues with this app, that’s gonna make me keep opening the app because there is nothing better than having information that is timed for when you need it or when you start looking for it. It’s almost like a reassurance that if I’m going through something at a certain time period, it’s probably going to be in that educational center at the time that I need it.

P2; nonbirthing parent; low app use

In terms of baby care, parents explained how the targeted content provided within the app helped them navigate the often overwhelming journey of early parenthood, fostering a sense of competence and security as they adapt to their new roles.

One participant reported the following:

The app in a way provided me with assurance that my baby is doing okay or that I am doing the right thing as a new parent. Sometimes I just want to know what to expect in terms of my child’s developmental milestones. The app works great because it shows you 6-9 months milestones and things to watch out for. I like reading ahead, because I’m a person that likes to plan. For example, after reading an article in the app about visual development and sleep regression, I contacted my pediatrician to discuss these stages in more detail and clarify any questions I had about this.

P8; birthing parent; low app use

Another parent commented the following:

There are times you go to Google to search for something and are overwhelmed with hundreds of articles where everyone says something different. All the articles on the B2H app are relevant, and I feel safe to follow the information provided by the app because I noticed that all the articles list out the source they are coming from at the bottom which makes it trustworthy.

P7; birthing parent; low app use

### Parent-Reported Outcomes Indicating App Support During the Psychosocial Care Journey

#### Theme 1: Supporting Parents in Proactively Identifying Mental Health Symptoms Through Self-Monitoring

##### Subtheme 1: Enhancing Health-Promoting Behavior Through the Integration of Multiple Touchpoints and Reminders With Mental Health Screenings

Participants reported an increased engagement and involvement with their mental health care due to the integration of B2H. According to them, this was a direct result of ongoing assessment and the integration of multiple touchpoints with mental health screenings, which played a crucial role in encouraging and sustaining positive health behaviors.

Participants reported that, unlike clinical settings where birthing parents typically have just one touchpoint for mental health screening during the 6-week postpartum visit, the B2H app provided parents with a continuous and proactive method for managing their mental health. The monthly mental health surveys, which are a part of the B2H intervention, encouraged ongoing self-monitoring and reflection, allowing parents to maintain a consistent awareness of their mental well-being.

One parent shared as follows:

There are times when I feel deeply connected and engaged with my mental health, but other times I find myself rushing through the monthly surveys, reassuring myself that everything is okay or dismissing any concerns. Yet, there are moments when certain questions prompt me to pause and reflect, often bringing me to tears and making me realize that I may indeed feel a certain way or need to pay closer attention to my mental well-being. These moments underscore the importance and value of consistently taking these surveys.

P19; nonbirthing parent; high app use

In addition, parents in the intervention group reported that the app’s reminders and prompts for regular check-ins contributed to a more structured and disciplined approach to mental health care.

One parent noted the following:

I am a big believer in staying on top of my mental health; however, there were times when I did not remember to open the app on my own. The in-app notifications, text reminders and care manager follow-ups, helped me ensure that I didn’t miss any screenings. There were certain times when the reminders were very helpful in reminding me to carve some time for myself and reflect on my mental health. It gave me the space to think rationally about whether I needed to have a conversation with my partner or make an appointment with a therapist.

P10; birthing parent; low app use

##### Subtheme 2: Improving Symptom Awareness as an Outcome of Remote Screening and Comprehensive Mental Health Assessments

Enhanced symptom awareness emerged as another theme in the qualitative interviews. According to the participants, this was a direct outcome of the integration of comprehensive mental health assessments (PHQ-9, GAD-7, and PSS) and remote screening.

Parents reported that the use of comprehensive, evidence-based screening tools enabled them to identify symptoms of anxiety or stress that they might have otherwise overlooked, especially if their initial screenings for PPD during their postpartum visits were negative.

One parent shared the following:

There have been stressful moments, certainly, but I haven’t felt like I had depression or anything along those lines, so I haven’t been actively seeking out help. However, seeing my screening results was truly eye-opening. Although my scores for PhQ9 were moderate, I had no idea that I was experiencing such high levels of stress and anxiety.

P5; birthing parent; low app use

In addition, parents who completed paper-based screenings during their postpartum visit reported that they often rush through the assessment, potentially overlooking critical questions about their own mental health as they prioritize asking questions about their infants during their postpartum visit.

One participant noted the following:

I felt like I was navigating this complex web of decisions on my own, without consistent guidance or support from my healthcare providers. I attended my 6-week postpartum visit and was scheduled for a 12-week one, but I did not attend it. I feel that the care offered to parents during pregnancy is much better than the postpartum care, which often feels neglected and rushed. In reality, all the support I received during my postpartum visit was mostly centered around caring for the newborns.

P16; birthing parent; high app use

On the other hand, parents who engaged with the B2H screenings reported that the transition from paper-based screening to using mHealth tools for mental health assessments provided them with the flexibility needed to manage their mental health more effectively and proactively. A substantial number

of parents reported that the flexibility and privacy of completing these assessments remotely at their convenience enabled them to take more time for self-reflection, which is often not possible in clinical settings.

One parent remarked as follows:

The mental health surveys provided by B2H are a nice way for me to reflect upon certain things at my own pace, allowing me to think more deeply about my feelings and recognize symptoms I might have previously missed. The digital surveys are also quick, like they only take about like five minutes or so. So it’s just those five to ten min where I get to focus on my own mental health. Outside of these surveys, I haven’t had much time to really worry much about my own well-being at this point.

P3; nonbirthing parent; high app use

##### Subtheme 3: Empowering Birthing Parents to Recognize and Respond to Mental Health Symptoms in Partners Through App Support

The qualitative study findings revealed that by providing a more inclusive approach that screens both birthing and nonbirthing parents, the app not only helped facilitate early detection of mental health issues but also created awareness among birthing parents on the potential mental health challenges their partners might face, which often go unrecognized:

For me and my husband, the ability to see our test scores and keep taking those surveys is valuable. Instead of having a pen and paper and having to calculate our scores, the app does a good job tracking our scores for us and is right there at your fingertips. When my husband or I get the survey reminders, we remind each other to take the survey and read the resources provided within the app. We would not compare our scores but discuss them and collectively reflect on the moments that may have resulted in elevated scores. When both you and your partner feel like you are at 25% and need to be there at 100% for the sake of the baby, having these surveys reminds us that we need to talk to each other about this and take time for ourselves or together or as a family if we’re both feeling stressed.

P12; birthing parent; high app use

Parents reported that the educational content provided on the B2H app equipped them with the knowledge needed to notice symptoms or signs of depression or anxiety in their partners. In addition, the articles helped parents support their partners by sharing useful strategies for managing symptoms and seeking professional help if needed, allowing for equitable mental health care for all parents, regardless of their role in birthing.

One birthing parent reported the following:

I specifically enjoyed reading the articles on how to communicate with your partner regarding fatigue and taking some time for yourself. There were times when the article would tell us what to look out for, what to avoid doing, and make suggestions for suitable alternatives, which were pretty helpful. Since my wife and I work in the mental health profession, we often tend to rationalize our mental health. So the articles are a great reminder for us to dedicate time for our self-care and check in on each other’s mental health.

P17; birthing parent; high app use

Another parent shared as follows:

I often felt reassured by my own screening results, but seeing my husband’s results was truly eye-opening. I had no idea he was experiencing such high levels of stress and anxiety, as he hides it so well. Without the monthly assessments, I would never have known about his struggles. Seeing his scores made me start checking in on him more frequently to see how he is doing and encourage him to actively seek out mental health support if needed.

P5; birthing parent; low app use

#### Theme 2: Timely Assessment and Rapport Building Through Care Manager Support

The qualitative study revealed that the presence of a CM to monitor scores made a significant difference in the experiences of parents. A significant number of participants reported that having a CM to monitor their scores and provide timely feedback markedly improved their overall experience and outcomes.

One parent reported the following:

Although I did not communicate with my care manager frequently. I appreciate that the option was given to me. The care manager reached out to me in those early weeks to let me know that she was available and was there to monitor our mental health scores based on the assessments. It was almost as if there was someone paying close attention to your mental health and even though our communication has not been frequent or consistent, I do know that they’re available, which is reassuring.

P15; birthing parent; low app use

Feedback from parents underscores the value of having a dedicated CM monitor their scores, compared to an app that lacks human oversight. Parents expressed that knowing there is a person on the other end tracking their progress makes them feel reassured that their mental health is being actively monitored by a professional, thereby strengthening their engagement and confidence in the care process. This human element offered parents a reliable touchpoint for ongoing support, contributing to a more personalized and effective care approach.

One participant reported as follows:

To me, having a care manager and monthly mental health surveys within the app goes above and beyond any other baby tracking apps. My care manager did a great job of reaching out to me and checking in with me regularly. She was very responsive and knew when to push and when to pull back. This way I could utilize her in a manner that worked well for me.

P4; birthing parent; high app use

#### Theme 3: Supporting Mental Health Treatment Through Care Coordination and Engagement With Mental Health Modules

When asked how the app supported treatment, many parents highlighted the crucial role of CMs in helping them navigate their treatment plans and the significant impact of the mental health modules in providing effective coping strategies. The combination of these elements fostered a sense of support and empowerment among parents, ensuring that they felt confident and prepared in managing their mental health.

One parent stated the following:

A few days after taking my assessment, a care manager reached out to me and offered to do the Behavioral Activation (BA) sessions with me. These sessions were easy to follow, providing tips and methods on how to effectively handle stress. This was incredibly helpful, allowing me to practice different ways of coping with anxiety and stress. The care manager also shared some articles in the chat feature of the app, which gave me something to go back and look into every time I feel anxious.

P7; birthing parent; low app use

Another parent emphasized the role of CMs in helping them feel supported with their treatment decision:

Having a care manager to discuss the risks and benefits of treatment options made me feel prepared and a bit more confident in my decisions. The conversations we had allowed me to dig deeper into the root causes of what was going on. The care manager would provide resources to support me and schedule check-ins with me. She would follow up kind of in between the survey periods to check and see if the resource she sent worked or if I needed additional support. I think just having that kind of ongoing support made me feel empowered and more likely to engage in my care. Plus the ability to reach out to her at any time gives me this added sense of security and makes me feel less isolated in my treatment journey.

P14; birthing parent; high app use

Parents also appreciated the CMs’ follow-ups to ensure that they remained connected to care and helped them find therapists covered under their insurance. One parent reported as follows:

While the app did not directly influence my treatment decisions, the follow-ups provided by care managers played a crucial role in helping me connect with a therapist. The care manager followed up with me regularly to check on my progress and make sure I was connected to the right care. The care manager even helped me find a therapist covered by my insurance, which was a huge relief.

P19; birthing parent; high app use

#### Theme 4: Empowering Parents With the Resources and Support Needed to Ensure a Smooth Transition Out of the CCP

Parents nearing the end of their postpartum care journey reported that the B2H intervention’s approach of integrating continuous support, educational resources, and follow-up care has empowered them to manage their mental health effectively, ensuring a smooth transition out of the CCP. This holistic support system has fostered a sense of confidence and preparedness among parents, allowing them to maintain their well-being independently.

The transition out of the CCP often presents challenges for parents. As parents draw near to the end of their postpartum care journey (12-month postpartum milestone), they usually find themselves needing to connect with external psychosocial care providers to ensure continuity of care. This transition can be fraught with difficulties, such as connecting with new therapists or finding a therapist covered by their insurance. The support provided by the CMs during this transition was particularly appreciated by parents, as it ensured that they remained connected to care resources and felt supported in their mental wellness journey.

One parent shared their experience, stating as follows:

I recently moved to a new state and have been struggling to find a therapist that is covered by my healthcare plan. I reached out to the care manager through chat hoping to seek recommendations for a therapist and a new primary care provider. The care manager not only provided me with several options but also followed up to ensure I had all the resources necessary to support the continuity of my care. Having the care manager check in regularly gave me a sense of continuity. I didn’t feel like I was being left on my own.

P17; birthing parent; high app use

Parents also shared the importance of the guidance and support provided by the educational articles, which helped them maintain the progress they had made during the program.

One parent highlighted how the knowledge gained from the app’s educational content prepared them for life after the CCP:

The articles and mental health modules on the app taught me so much about recognizing and managing my symptoms. The articles on identifying potential triggers and the ones on stress management techniques gave me the tools needed to confidently handle any future challenges. I now feel equipped with resources needed to help me keep up with my mental health, making it less intimidating to handle things on my own once my postpartum journey ends.

P9; birthing parent; high app use

### B2H’s Role in Addressing Gaps in Usual Care at the Intersection of the 2 Journeys

#### Overview

In addition to demonstrating how the B2H app effectively supports the comprehensive needs of parents in their postpartum care journey, our findings also illuminate several critical gaps in usual care as reported by the parents. By applying the PJF to our analysis, we were able to uncover how these gaps manifest in the real-world experiences of parents and explore the potential of mHealth technology to address these deficiencies.

**Figure 6:**
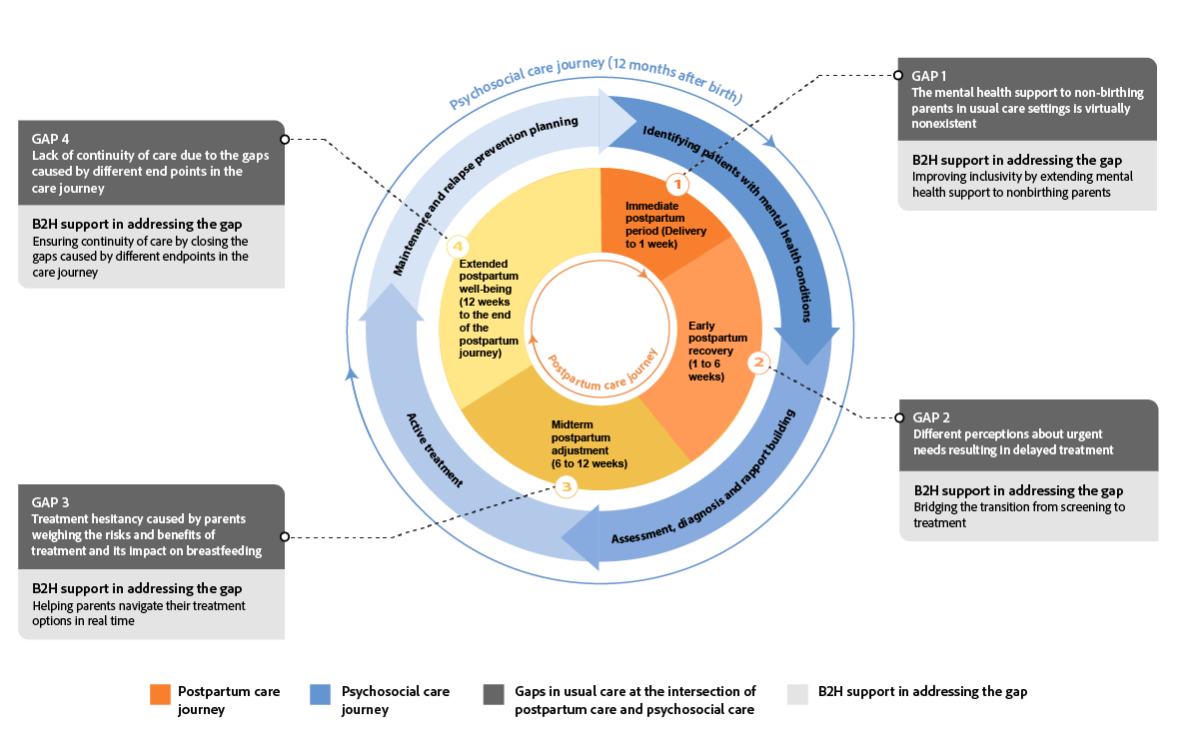
Gaps at the intersections between the psychosocial care journey and the postpartum care journey.

At the intersection of the 2 journeys, we provide the gaps identified by our data, noting that additional unobserved gaps may exist. Figure 6 demonstrates these gaps and highlights the role of mHealth technology in closing these gaps.

#### Improving Inclusivity by Extending Mental Health Support to Nonbirthing Parents

The interview findings revealed that the mental health support provided for nonbirthing parents in usual care settings is virtually nonexistent.

One parent reported the following:

When I first logged into the app, I just assumed that the app was mainly for baby tracking. That’s kind of the only way I was thinking about it. I think as new dads you often get conditioned to thinking that most of the resources and support out there are mainly for the moms or the baby. Sadly dads often get overlooked when it comes to mental health support. Besides the mental health surveys on the apps I don’t think I have had any other encounter with mental support in the real world.

P2; nonbirthing parent; high app use

This finding supports the existing literature, which indicates that the current system lacks any framework to support screening or treatment for nonbirthing parents, highlighting a significant oversight in the existing approach to care [44]. Unlike routine assessments for depression and anxiety symptoms in new birthing parents during postpartum visits, there is a notable absence of similar checkups for nonbirthing parents. This exclusion often hinders nonbirthing parents from actively participating in postnatal care and fully understanding their role in supporting both the birthing parent and the newborn.

Nonbirthing parents in the intervention group reported positive experiences with the B2H intervention, noting how the tools and resources provided within the app empowered them to take charge of their own mental health and actively participate in the care of their partners. This involvement not only enhanced their own well-being but also contributed to a more supportive and collaborative postpartum care environment.

A nonbirthing parent stated as follows:

My wife was dealing with some health stuff before this. So every time she fills out the survey, the care managers in the app check in with her which is nice because there is someone besides me looking out for her. That being said, we are now a little bit aware of our scores due to the assessments and try to track and manage each other’s mental health.

P13; nonbirthing parent; low app use

In addition, the app also enabled nonbirthing parents to take a proactive role in supporting their partner’s mental health journey. It provided them with the tools to regularly check in on their partner’s mental well-being, engage in shared decision-making, and offer timely encouragement for seeking professional help when necessary.

One parent recounted the following:

Four months into having the baby my husband and I were having a lot of stress mainly because of him working late hours and my health. We did not know how to communicate what we were feeling. Taking the surveys in a way helped us communicate better about our scores and my partner suggested for us to go back to therapy as a way of dealing with our frustrations.

P10; birthing parent; low app use

The findings of this study also underscore the role of mHealth technology in facilitating communication and engagement within the family unit. Nonbirthing parents reported that using the B2H app helped them become more informed on critical aspects of postpartum care, including postpartum visits, contraception, and breastfeeding. This increased knowledge significantly enhanced their involvement in their partner’s postpartum care journey, enabling them to effectively communicate the importance of attending postpartum visits, encourage continued breastfeeding, and provide both emotional and practical support.

One parent explained as follows:

My husband reads more of the articles that I do and informs me on what he reads which in turn helps me stay informed. If my partner comes across an interesting article on the app he shares it with me and after we talk about it for a bit I use Google to dig deeper and get more information surrounding the topic. Like the other day he read something about questions to ask during the postpartum visit and shared the article with me.

P6; birthing parent; high app use

However, it is important to note that although nonbirthing parents in the intervention group regularly engaged in the monthly assessments, their app use indicated limited interaction with the mental health modules and low chat use. Further research needs to be conducted to identify ways to enhance the engagement of nonbirthing parents with the CM and the mental health modules.

One parent reported the following:

I do not think I reached out to the care manager as much as she reached out to me to check in with me and see how I am doing. She reached out to me based on my scores and shared articles with me. I feel like, just because I read all the articles shared by my care manager I do not really feel like I need to approach her. We would chat like once in a while, especially in the beginning. I do not really need to go to my care manager just because I feel like the app offers a lot of support already.

P3; nonbirthing parent; high app use

Another parent reported the following:

I personally have not engaged with the mental health content. There’ve been stressful moments, certainly, but I haven’t felt any sort of depression or anything along those lines. I see therapists on the side so I didn’t use the app for any mental health content. I actually do teletherapy through zoom but occasionally meet in person. But you know, because I see a therapist monthly I don’t normally seek things like this on an app.

P19; nonbirthing parent; high app use

#### Bridging the Transition From Screening to Treatment

Another gap identified by parents in our study was the difference in perceptions between health care providers and parents regarding what constitutes urgent needs, particularly in the context of mental health.

One parent explained as follows:

I have a history of depression and anxiety, I was really hoping my postpartum visit would address my mental health more seriously. Instead, I felt like my concerns were kind of brushed off by my provider. The OB told me it was just the typical stress and anxiety that new moms experience. It didn’t feel like my needs were prioritized at all, and I left feeling like I wasn’t really heard or understood.

P7; birthing parent; low app use

This gap often exacerbates challenges, especially during the critical transition from screening to treatment. While national guidelines emphasize the importance of screening for conditions such as PPD and anxiety, the actual follow-through from an elevated screening result to a formal diagnosis and subsequent treatment is often faced with obstacles. These challenges arise because the importance of addressing mental health concerns is not always uniformly recognized by all key informants in usual care settings. Obstetric clinicians, who do not have specialized training in mental health, might underestimate the urgency of the patient’s mental health needs. In contrast, parents experiencing significant distress may perceive their needs as urgent but find that the health care system’s response does not align with this urgency. This misalignment can lead to delays in care, underdiagnosis, and, ultimately, untreated mental health conditions.

mHealth technology offers a promising solution to bridge this perception gap and facilitate a smoother transition from screening to treatment. Parents reported that the ability of mHealth technologies to track and monitor real-time data allows both parents and CMs to identify any changes in the mental health status and alert them if a situation requires immediate attention. Parents in the study also mentioned that the use of standardized screening tools, such as the PHQ-9, GAD-7, and PSS, empowered them to regularly assess their mental health and communicate their needs and concerns more effectively, making it less likely that their symptoms would be overlooked.

One parent reported the following:

Everyone should be screened for depression and anxiety during the postpartum visit. However, what actually occurs can be quite different. While many screen for postpartum depression, only a few are beginning to screen for anxiety, despite the fact that perinatal anxiety is incredibly common. Having these monthly reminders and surveys supported me by highlighting any red flags or providing resources to help me or like point me in the right direction. Although some of the questions in the survey were not necessarily applicable for me, I still think it’s important for people who may have a change in their relationship status or might not have the support system that I have. For parents who respond to surveys indicating a change in their relationship or health with the app I would assume that the app would then allow the care manager to reach out, check in with them and make sure they have additional support.

P17; birthing parent; high app use

Moreover, mHealth apps, such as B2H, can streamline the transition from screening to treatment by automatically flagging positive screening results for further evaluation by dedicated CMs. These CMs, trained specifically to identify and respond to symptoms of mental health issues, use a triage model to assess the severity and urgency of the cases. This approach ensures that parents with urgent needs receive immediate attention, reducing their reliance on obstetric clinicians to make diagnostic decisions outside their expertise and ensuring timely and appropriate referrals for those at elevated risk.

Another parent stated as follows:

Having a care manager was reassuring during my early days as a parent. It made me feel less alone, knowing there was someone there to check in on me and track my progress. As a new parent, you’re always questioning yourself: Am I doing this right? Is this experience normal? Everyone’s journey is different, so having a care manager to reassure me that I was on the right track made a huge difference. I think my care manager reached out to me right after my first mental health survey when we were in the NICU, and my stress levels were high as we tried to navigate everything. In a way the care manager made me feel validated, and validated my feelings more than anything.

P4; birthing parent; high app use

These findings underscore the benefit of integrating mHealth technology into the postpartum care process, particularly in enhancing the triage system and enabling CMs to track symptom scores in real time. However, the success of this approach relies on the presence of trained collaborative care coordinators within the mHealth framework, who are essential for monitoring changes in parents’ mental health status as they occur. By combining real-time data collection with expert analysis, CMs can respond swiftly and accurately to emerging needs. This synergy between technology and specialized care ensures that timely interventions are delivered, effectively bridging the gap between screening and treatment and providing parents with the support they need exactly when they need it most.

#### Helping Parents Navigate Their Treatment Options in Real Time

Our findings indicated that parents who engaged in mental health treatment often had to consider not only their psychosocial well-being but also their obstetric and infant care journeys. This multifaceted decision-making process involved weighing advice from multiple health care providers and evaluating the potential impacts of treatment—especially medication—on breastfeeding and overall infant health. For some parents, this created a significant source of anxiety, often delaying treatment decisions or causing parents to question the appropriateness of certain interventions.

A major concern expressed by parents was the potential impact of treatment, particularly medication, on breastfeeding.

One parent shared the following:

I have a history of ADHD [attention deficit hyperactivity disorder] and was taking medication prior to my pregnancy. But ever since I had the baby, I stopped and haven’t been actively seeking out medication since I was still breastfeeding and wasn’t sure if it would affect my baby and whether it was safe.

P7; birthing parent; low app use

This uncertainty reflects the lack of preparation and support provided to parents once they leave the hospital after delivery. While health care providers typically offer some guidance on postpartum care during the hospital stay, this support is often minimal, leaving parents to navigate the complexities of postpartum care on their own.

One parent noted as follows:

There was so much information given to me at the time of delivery, but as soon as I got home, I realized how little I actually knew about managing postpartum care, especially when it came to breastfeeding while considering treatment for my mental health. It felt like I was on my own.

P9; birthing parent; high app use

Parents in this study recounted how the abundance of online resources, while well-intentioned, often became overwhelming. The sheer volume of information left many feeling more confused and anxious than reassured.

One parent described as follows:

I spent hours researching what is safe during breastfeeding. We have a pile of books in my house that we’ve tried to make our way through, but have been looking for quick little snippets or tidbits of valuable information that you can quickly read and digest in comparison to reading 300-400 pages. I tried googling this information but every website seemed to have conflicting advice, and I wasn’t sure who to trust.

P5; birthing parent; low app use

This overwhelming influx of information further highlighted the gap in accessible, timely support during the postpartum period. Without clear, reliable guidance from health care professionals, parents were left navigating complex decisions about their mental and physical health alone, often unsure of the best course of action for their postpartum journey.

The findings from this study underscore the critical role that CMs can play in helping parents make these complex decisions. Care managers, by providing personalized support and guidance, could alleviate much of the confusion and anxiety of parents that stems from the sheer volume of information they encounter. Parents reported how CMs serve as a reliable, accessible resource for answering questions and helping parents navigate their treatment options in real time. This proactive approach to addressing concerns about breastfeeding and mental health treatment can help parents feel more confident in their decision-making.

One parent reflected on how having a CM made a difference in her postpartum care journey:

I was unsure about starting medication while breastfeeding. My care manager was the one who really took the time to answer all my questions and made sure I had the latest information from trustworthy sources. It felt reassuring to have like a medical professional guide me through the process, and I didn’t feel as anxious about making the decision anymore. Without her, I would have been lost in all the information I found online.

P9; birthing parent; high app use

These findings highlight the complex decision-making process parents face when managing their postpartum mental health. The lack of consistent, accessible support after hospital discharge leaves parents vulnerable to confusion and anxiety, often causing treatment hesitancy. The inclusion of a CM within the app provided parents with an additional layer of support, offering personalized, timely support needed to make informed decisions about their mental health and breastfeeding, ultimately promoting more effective treatment engagement. This organized and responsive system of care ensures that parents are not left to troubleshoot issues on their own but are supported in a way that is both comprehensive and accessible. By doing so, mHealth transforms the experience of postpartum care from one associated with feelings of uncertainty and being overwhelmed to one that fosters empowerment and encourages proactive self-care.

#### Ensuring Continuity of Care by Closing the Gaps Caused by Different End Points in the Care Journey

In most usual care settings, the obstetric care journey comes to an end with the postpartum visit that is usually scheduled during the early postpartum recovery phase (approximately 6 weeks after birth). However, the psychosocial care journey extends 12 months after birth. This disjointed timeline creates challenges for psychosocial care providers and impedes the patient’s ability to receive continuous and appropriate care during a critical period when they may still be experiencing or developing mental health issues, such as PPD.

As regular contact with obstetric care providers diminishes after the 6-week postpartum visit, the window for ongoing monitoring and early intervention for issues such as depression and anxiety narrows significantly. This gap creates a vulnerability in the health care system, where the absence of sustained medical oversight may allow psychosocial challenges to go undetected and untreated. The mismatch between the end point of the obstetric care journey and the continued need for psychosocial support highlights the need for more integrated and continuous care pathways that extend beyond the initial postpartum period.

One parent highlighted this gap and reflected on her postpartum journey, stating as follows:

It’s kind of sad to think about the existing state of postpartum care. I definitely feel like there is so much more that can be done to support new birthing parents with their postpartum journey. There’s a striking gap in the time and resources allocated towards pregnancy versus the postpartum period. While a woman might visit her OBGYN multiple times during pregnancy, she’ll typically only visit the OB once or twice during the postpartum period. And while it’s common for an expectant birthing parent to take a birth education class, it is virtually unheard of for her to receive postpartum education.

P20; birthing parent; high app use

mHealth technology offers a promising solution to bridge this care gap by providing continuous, accessible support throughout the extended postpartum period. Through mHealth apps such as B2H, new parents can maintain regular engagement with CMs even after the formal end of their obstetric care. The findings of our study indicate that integrating a CM within mHealth technologies helps facilitate ongoing monitoring of mental health through self-assessments and screening tools that can be administered at intervals well beyond the 6-week mark, ensuring that symptoms of depression or anxiety are not overlooked as time passes.

One parent explained as follows:

I really assumed postpartum was only like the first few months after having a baby and I’m learning that that’s not the case at all. Now I understand that postpartum doesn’t necessarily have a finite time. The surveys kind of prompted me to reconsider what I thought the timeline was for postpartum. What I found most helpful was the care manager check-ins from time to time. I liked bouncing ideas off the care manager. Just having a conversation with the care manager solidified what I was thinking. What I liked about the chat feature was the ability to touch base with someone because you can feel very isolated when you’re home with the baby all day.

P11; birthing parent; low app use

In addition to facilitating continuous monitoring, integrating various support resources, such as educational content, mental health assessments, and a chat functionality, helps maintain a connection between the patient and their care coordinator, promoting a more seamless transition from obstetric care to comprehensive psychosocial support.

Another parent compared the B2H app to other existing apps, stating the following:

There are a lot of apps that once you sign up, they’ll send you emails or reminders about upcoming milestones and things like that. However B2H has a lot more daily education that keeps you engaged for the entirety of the postpartum journey. I think with this app there’s a lot of information there about things like, mindfulness resources. Just taking those surveys and knowing my scores were a great indicator of my mental health. As a new parent you assume that after six weeks or twelve weeks things are supposed to get better and your scores should be improving but that is always not the case. I did feel that especially after the last survey which prompted me to reflect on the month that I had and what I need to do differently in the next month so that my scores are lower such as looking for a postnatal counseling services.

P13; birthing parent; low app use

By leveraging mHealth technology, health care providers can ensure that the psychosocial care journey is not abruptly interrupted by the end of the obstetric journey. Instead, these tools create a continuum of care that supports new parents throughout the first year after birth. This approach not only enhances the ability to monitor and manage mental health concerns but also ensures that the support offered is consistent, responsive, and aligned with the evolving needs of the patient.

## Discussion

### Principal Findings

#### Evolving Postpartum Needs and the Role of mHealth

##### Summary of Key Findings

A summary of key findings about evolving postpartum needs and the role of the mHealth app examined in this study is provided in Table 1.

**Table 1:** Key findings about the evolving postpartum needs and the role of the mobile health (mHealth) app.

Phases in postpartum care	Emerging needs	App features used	Role of mHealth app (B2H)
Immediate postpartum phase	Navigating uncertainties related to their recovery and newborn care	Heavy reliance on the baby tracking feature (feeding, pumping, diaper, and sleep tracking)	Helped parents monitor newborn activities, support daily caregiving decisions, and reduce anxiety in early care routines
Recovery phase	Establishing routines, enhancing communication with health care providers, and sharing caregiving responsibilities with partners	Continued use of shared baby tracking and educational content (eg, breastfeeding and infant safety)	Facilitated partner collaboration, supported routine formation, and prepared parents for well-child visits and postpartum visits
Midterm postpartum phase	Shifting focus to parental well-being and self-care	Educational content on self-care and monthly mental health assessments	Supported fatigue management, hormonal balance, and mental and emotional health through timely, relevant guidance
Extended postpartum phase	Supporting the baby’s developmental milestones	Educational content on baby development	Helped parents track developmental progress and provided age-specific guidance to support caregiving decisions

##### Implications for mHealth Design

The findings of our study underscore the importance of designing mHealth interventions that can dynamically adapt to the evolving needs of parents across the postpartum journey. In the early and recovery phases, parents exhibited heavy reliance on the baby tracking features (feeding, pumping, and diapering). However, usability issues such as inability to save entries due to app session timeouts, laggy response times when inputting data, and lack of visual clarity in the time tracking interface resulted in decreased app use, prompting parents to switch to alternative apps that provided similar tracking functionalities. Parents also expressed a desire for more personalized visualizations of daily activities and trends across days and weeks to help make sense of their baby’s patterns—features available in competing apps. These specific pain points influenced app switching behavior not because users found the app irrelevant, but because they perceived the core tracking functions as unreliable or inefficient. This highlights the importance of seamless design and usability to maintain engagement, particularly when parents need reliable tools the most. In mHealth apps, where user engagement is tied directly to the ability of the app to meet evolving and time-sensitive needs, a frictionless experience is essential. Frustration with core features can lead to disengagement, reduced intervention effectiveness, and fragmented care, limiting opportunities for holistic support such as mental health check-ins and educational content.

As parents progressed into the midterm and extended postpartum phases, their needs shifted toward self-care, mental well-being, and baby developmental milestones. The B2H app’s integration of educational content on postpartum recovery and mental health proved particularly valuable during this transition. To better meet these changing needs, future mHealth interventions should consider incorporating modular designs that adjust based on the stage of parenthood. In addition, personalized content recommendations based on the baby’s developmental stage and parental mental health screening results could help ensure that mHealth tools remain relevant and engaging throughout the postpartum journey.

#### Addressing Psychosocial Needs With mHealth Support

##### Summary of Key Findings

A summary of key findings about addressing psychosocial needs with mHealth support is provided in Table 2.

**Table 2:** Key findings about addressing psychosocial needs with mobile health (mHealth) support.

Phases in psychosocial care	Emerging needs	App features used	Role of mHealth app (B2H)
Identification of patients with mental health conditions	The need to enhance health-promoting behavior, improve symptom awareness, and empower birthing parents to recognize symptoms in their partner	Monthly mental health assessments using PHQ-9^a^, GAD-7^b^, and PSS^c^	Helping parents manage their mental health by offering multiple assessment touchpoints throughout the postpartum journey Enabling early detection through the use of comprehensive, evidence-based screening tools Providing remote, self-paced screening, giving parents more time and comfort to reflect on their mental health Enhancing symptom awareness, including recognition of mental health challenges in their partners
Initial psychosocial assessment, diagnosis, and rapport building	Timely assessment and rapport building through care manager support	In-app chat functionality	Enabling parents to receive timely support and personalized guidance in managing their mental health by assigning them a dedicated care manager
Active depression treatment	Supporting mental health treatment through care coordination and engagement with mental health modules	Mental health education modules (such as behavioral activation module and stress module); follow-up from care managers	Helping parents actively manage depression, anxiety, and stress by tracking their mental health score progression and providing them ongoing access to mental health modules and resources Facilitating informed decision-making by allowing parents to discuss treatment options with a care manager Offering care manager support to help parents connect with therapists or additional services, improving access to professional care
Maintenance and relapse prevention planning	Empowering parents with resources and support for a smooth transition out of the collaborative care program	Long-term postpartum care resources; care manager–supported transition guidance; reminders and check-ins on relapse indicators	Helping parents sustain the progress made during the program and prevent relapses through educational resources and articles Supporting continuity of care by helping parents connect with external psychosocial health care providers as they approached the 12-month postpartum milestone Providing care manager guidance during the transition period to ensure that parents remained supported and aware of available resources beyond the conclusion of the collaborative care program

^a^PHQ-9: 9-item Patient Health Questionnaire.

^b^GAD-7: 7-item Generalized Anxiety Disorder.

^c^PSS: Perceived Stress Scale.

##### Implications for mHealth Design

The B2H intervention’s success in addressing psychosocial needs demonstrates the ability of mHealth tools to enhance postpartum care. The continuous touchpoints for mental health assessments, combined with CM support, played a crucial role in fostering health-promoting behaviors and ensuring timely mental health interventions. Furthermore, the combination of automated features, such as reminders and surveys, with personalized CM support highlights the importance of integrating both digital and human elements in mHealth solutions. This hybrid approach creates a balanced system where technology facilitates self-monitoring while CMs provide tailored support, ensuring a more holistic and personalized experience.

However, the findings also highlight the need for more targeted efforts to engage nonbirthing parents and address the unique psychosocial challenges they face. This study revealed disparities in CM engagement between birthing and nonbirthing parents. Nonbirthing parents reported lower interaction levels, which may have limited the intervention’s effectiveness for them. Although less involved in physical recovery, nonbirthing parents face significant psychosocial challenges and report elevated stress and anxiety. Their reduced engagement suggests that they may not be receiving adequate support to manage these challenges. This gap highlights a key area for improvement in future iterations of B2H. Increasing outreach and tailored support for nonbirthing parents could enhance the app’s impact. Because birthing parents often influence their partners’ treatment decisions, educating them to recognize mental health symptoms in their partners and encouraging CM engagement could promote a more collaborative approach, ensuring both parents receive the support they need.

This calls for more equitable support across all users, regardless of their role in caregiving. Future mHealth interventions should consider developing targeted strategies to increase the engagement of nonbirthing parents. This could include personalized check-ins, more targeted reminders, a gamified approach, and additional resources focused on the unique psychosocial stressors nonbirthing parents face. In addition, providing educational content that encourages birthing parents to monitor and support their partners’ mental health could foster a more collaborative and inclusive approach to postpartum care.

#### Contribution of the PPJF in Highlighting the Holistic Needs of New Parents

The application of the PJF in this study provided a comprehensive approach to understanding the distinct yet interconnected experiences of both birthing and nonbirthing parents during the postpartum period. By mapping the postpartum and psychosocial care journeys of each parent and identifying the unique challenges they face, this framework allowed for a more nuanced understanding of how mHealth interventions, such as B2H, can support different roles within the caregiving unit.

CCPs have made significant progress in acknowledging the association between psychological and physiological challenges encountered by birthing parents, highlighting the growing awareness and importance of holistic care. Despite the increased attention to comprehensive patient care, 78% of women who screen positive for PPD do not receive mental health treatment, while nonbirthing parents receive little to no support when it comes to psychosocial care [4]. By applying our modified version of the PPJF to our study, we highlight several missed opportunities or gaps in usual care settings that resulted in parents receiving less-than-ideal care. Through the lens of the parallel journey, our work exposes many of these gaps at the intersection of postpartum and psychosocial care while drawing focus on technological opportunities to ensure that new parents receive adequate support and care.

One of the key contributions of this framework is the ability to draw attention to the often-overlooked psychosocial challenges that affect both birthing and nonbirthing parents. Our findings illuminated the gaps in usual care, particularly in how mental health support is disproportionately provided to birthing parents, while nonbirthing parents are left without adequate resources to manage their own mental health. As revealed in the interviews, nonbirthing parents frequently assumed their role was limited to supporting the birthing parent and the newborn, overlooking their own need for psychosocial support. This study showed how an mHealth intervention, such as B2H, can fill this gap by extending mental health resources to nonbirthing parents, encouraging their active involvement in caregiving and in their own well-being. The inclusion of nonbirthing parents in mental health support systems reflects a more inclusive approach to postpartum care.

In addition, the PPJF exposed several systemic issues that result in less-than-ideal care for parents. First, the findings revealed that health care providers and parents often have differing perceptions of what constitutes urgent mental health needs. This misalignment frequently results in delayed treatment, as mental health concerns raised by parents are sometimes dismissed or minimized by obstetric clinicians who may lack specialized training in mental health. By identifying this gap, the framework highlights the need for better alignment between parent-reported mental health concerns and health care provider responses, an area where mHealth technologies such as B2H can help by flagging urgent needs based on real-time assessments. Second, the framework also illuminated the issue of treatment hesitancy, particularly among birthing parents who face the challenge of weighing the risks and benefits of mental health treatment in the context of breastfeeding. Parents often hesitate to seek treatment, concerned about the potential impact on their infant’s health. The PPJF underscores the importance of providing clear, reliable guidance on how to balance mental health needs with infant care. mHealth interventions play a critical role here by offering educational tools and CM support to guide parents through these decisions, thus reducing anxiety and improving treatment engagement. Third, one of the most significant gaps identified through this study is the lack of continuity of care due to the different end points in obstetric and psychosocial care. While postpartum visits typically conclude around 6 weeks after birth, psychosocial care needs often extend much longer, leaving many parents without adequate support during a critical period of their mental health journey. The framework allowed us to highlight how mHealth technologies such as B2H can bridge this gap, providing continuous engagement and monitoring well beyond the obstetric care. This ongoing support ensures that mental health concerns, such as PPD or anxiety, are not overlooked as time passes.

Using the PPJF, we were able to illustrate the experiences of both birthing and nonbirthing parents as part of an interconnected system of care, where psychosocial and physical needs must be addressed simultaneously. It highlights the importance of integrating mental health screening and support into the postpartum journey as a continuous, rather than episodic, process.

### Limitations of This Study

While our findings provide valuable insights into postpartum care and have the potential to inform future interventions, several limitations should be acknowledged.

Selection bias is a notable limitation, as the experiences and engagement levels of participants who chose to enroll in the study might differ from those who declined participation. This could influence the generalizability of our findings, as those who opted in may have had a greater interest in or need for the support provided by the B2H app.

Another limitation is the relatively small number of nonbirthing parents (4/20, 20%) who participated in the study. This smaller sample size may not fully represent the diverse needs and feedback of all nonbirthing parents, which could lead to an incomplete understanding of how the app addresses their specific concerns during the postpartum period. Further research focusing specifically on nonbirthing parents is crucial to better understand their unique challenges and engagement with mHealth tools. By exploring their experiences in greater depth, future studies can identify opportunities to enhance app features and address any unmet needs, ultimately improving support for nonbirthing parents during postpartum care.

Furthermore, our study did not fully address the needs of postpartum parents without smartphones or those who may face barriers to accessing digital health interventions. To ensure greater inclusivity, our next steps include conducting a study in Kenya to explore how B2H can become more equity focused. This study will investigate modifications to the app that make mHealth tools accessible to parents across different socioeconomic and technological divides, ensuring that the benefits of digital health interventions reach a broader and more diverse population of postpartum parents.

Finally, the qualitative nature of this study, while providing in-depth insights, may limit the ability to generalize findings across larger populations. Future research could benefit from a mixed methods approach that combines qualitative insights with quantitative data to validate and expand upon these findings. By acknowledging these limitations, we hope to encourage further exploration and refinement of mHealth technologies, ensuring that they are inclusive, accessible, and capable of addressing the diverse needs of all postpartum parents.

### Conclusions

The implications of applying the PPJF to mHealth interventions are significant for both the design and evaluation of postpartum care technologies. This framework provides a structured way to understand how mHealth solutions can support multiple stakeholders within the caregiving unit, ensuring that both birthing and nonbirthing parents are engaged and supported throughout their postpartum journey.

From a design perspective, mHealth technologies should be developed with the recognition that postpartum care is not a singular experience but a set of parallel journeys that intersect and influence one another. This means that features such as mental health screening tools, educational resources, and CM support should be tailored to address the needs of both parents, with attention to how their roles and experiences differ and overlap. For nonbirthing parents, interventions should prioritize increasing their engagement with psychosocial care, helping them to navigate their role in both their own well-being and that of their partner.

In terms of evaluation, the PPJF offers a method for analyzing the success of mHealth technologies by examining their impact on the holistic care experience. It allows researchers and developers to measure not only the physical health outcomes of birthing parents but also the psychosocial outcomes for both parents. This approach can provide a more comprehensive understanding of how mHealth interventions improve care coordination, communication, and mental health outcomes for the entire family unit. By identifying gaps in the usual care settings and showing how mHealth technologies can address these gaps, this framework can be used by other researchers in the future design and evaluation of technologies for postpartum care as a way of ensuring that parents receive the comprehensive, continuous support they need during one of the most critical periods of their lives.

## Appendix

Multimedia Appendix 1 Baby2Home application features.

Multimedia Appendix 2 Summary of the participants and their app use.

Multimedia Appendix 3 Interview guide.

## Abbreviations

B2H: Baby2Home
BHP: behavioral health care provider
CCM: collaborative care model
CCP: collaborative care program
CM: care manager
GAD-7: 7-item General Anxiety Disorder
PHQ-9: 9-item Patient Health Questionnaire
PJF: parallel journeys framework
PPD: postpartum depression
PPJF: postpartum parallel journeys framework
PSS: Perceived Stress Scale
RCT: randomized controlled trial
