# Supplementary material for: Understanding the Implications of mHealth Technology in Collaborative Care Programs and Its Role in Supporting Postpartum Care: Qualitative Interview Study of the Baby2Home Intervention Using the Parallel Journeys Framework
Source: JMIR Pediatr Parent. 2025 Aug 26;8:e70936. doi: 10.2196/70936 (PMC12421202; doi:10.2196/70936)
Supplement: Multimedia Appendix 1 [file pediatrics_v8i1e70936_app1.docx]

## **Appendix A**

### Baby2Home Application Features (https://baby2home.com)

| **Features** | **Summary** | **Screenshots** |
| --- | --- | --- |
| Infant Wellness Resources | • Infant care trackers to  help boost parenting confidence/ competence  • Tracking feature for feeding, pumping, sleep, diaper, growth and important health information such as vaccination and medications.  • Notes feature that enables parents to create a checklist of questions for the healthcare provider.  • Shared tracking feature between partners to make it easier to parent together. | 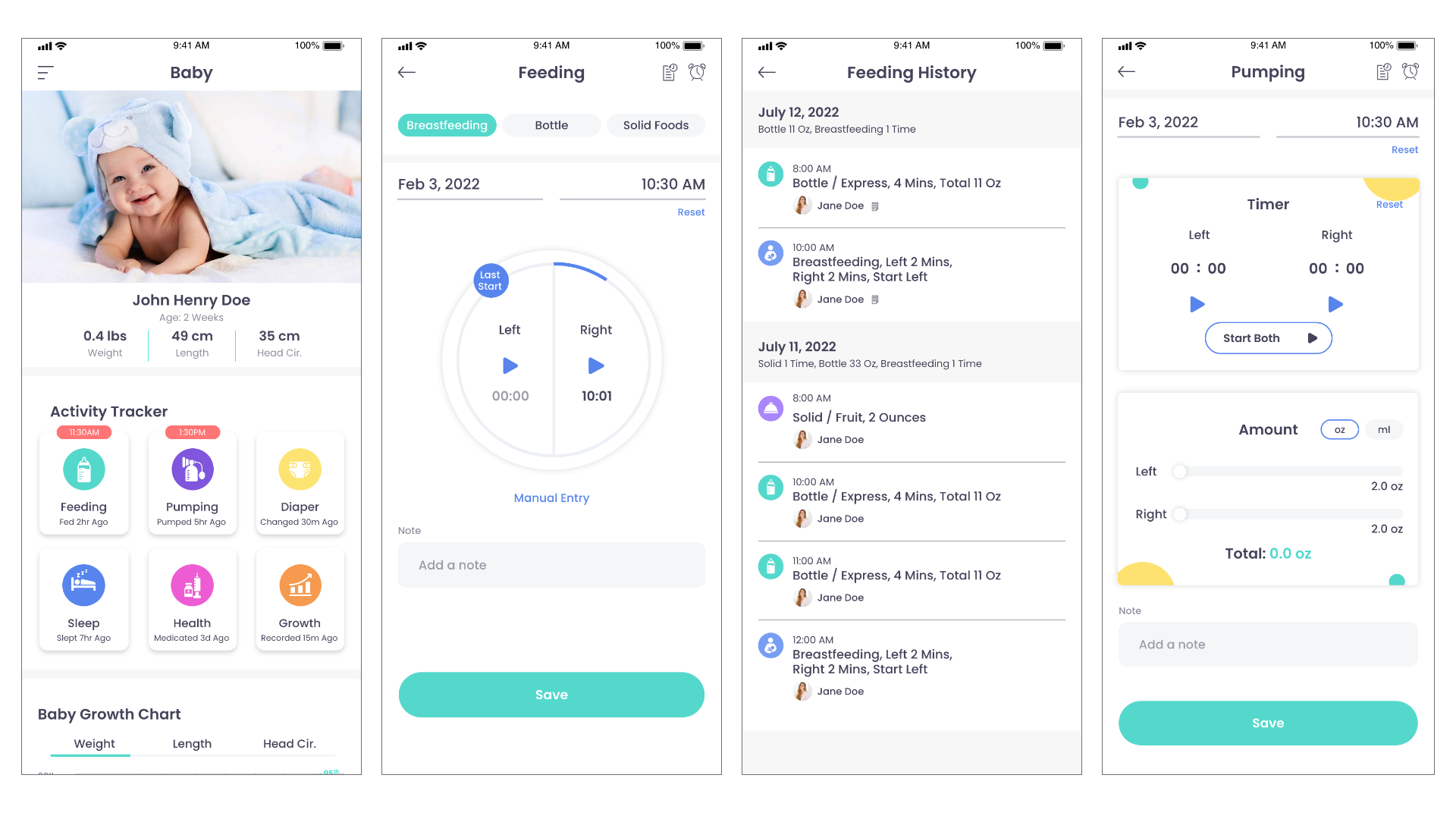 |
| Parental Education | • Library with over 250 articles on topics surrounding baby care, developmental milestones, parenting, challenges new parents face, self-care tips, physical and mental health and wellness resources.  • Anticipatory/timed educational content such as articles and videos tailored to the baby’s age and unique parenting journey. | 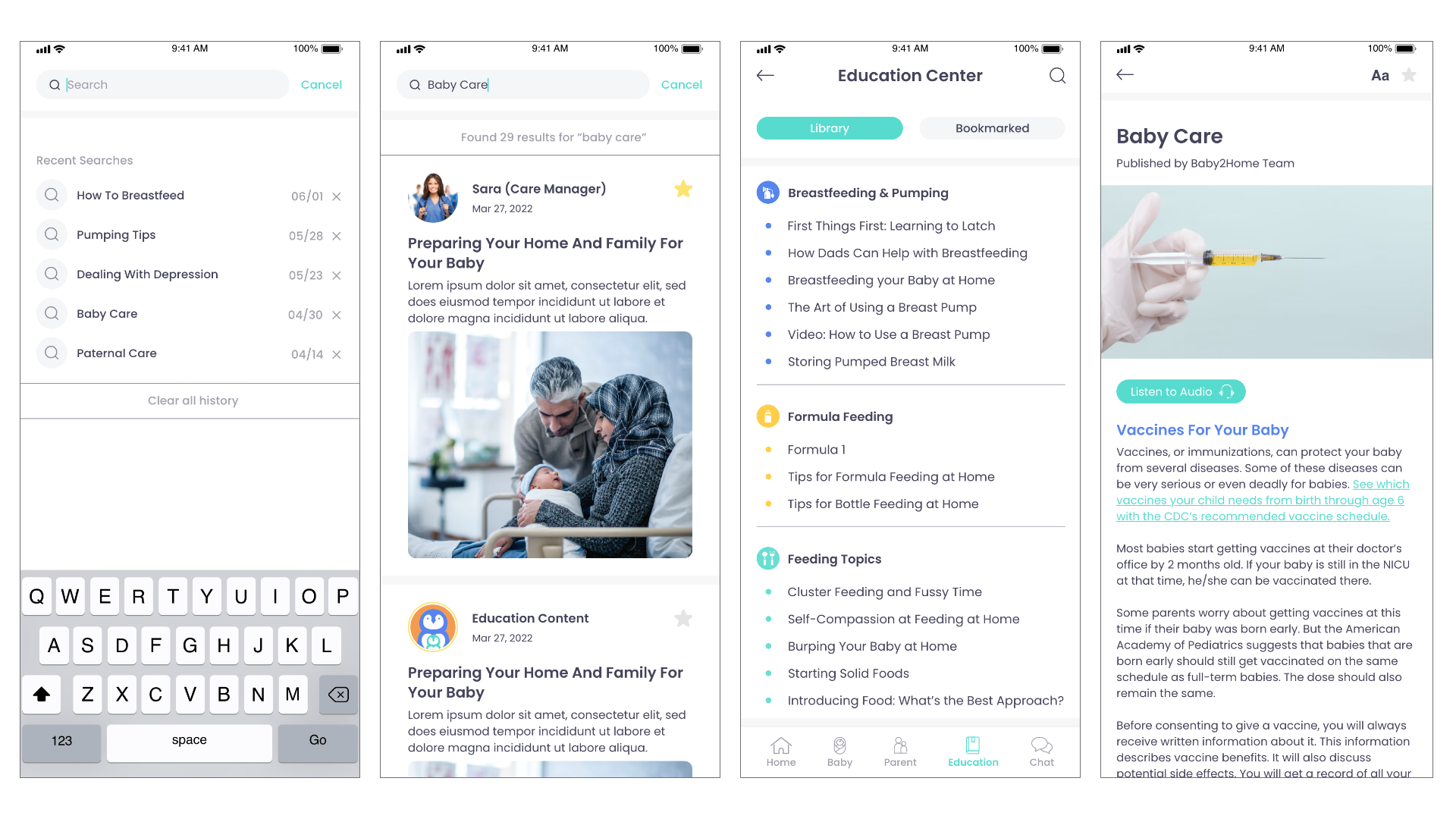 |
| Mental Health Screening & Support | • Monthly assessments and screening using standardized survey tools such as PHQ9, PSS and GAD7.  • Reminders and notifications to complete monthly screenings  • Screening results in the form of score progression report  • Access to mental health modules such as Stress Management and Resiliency Training (SMART) and Behavioral Activation (BA) | 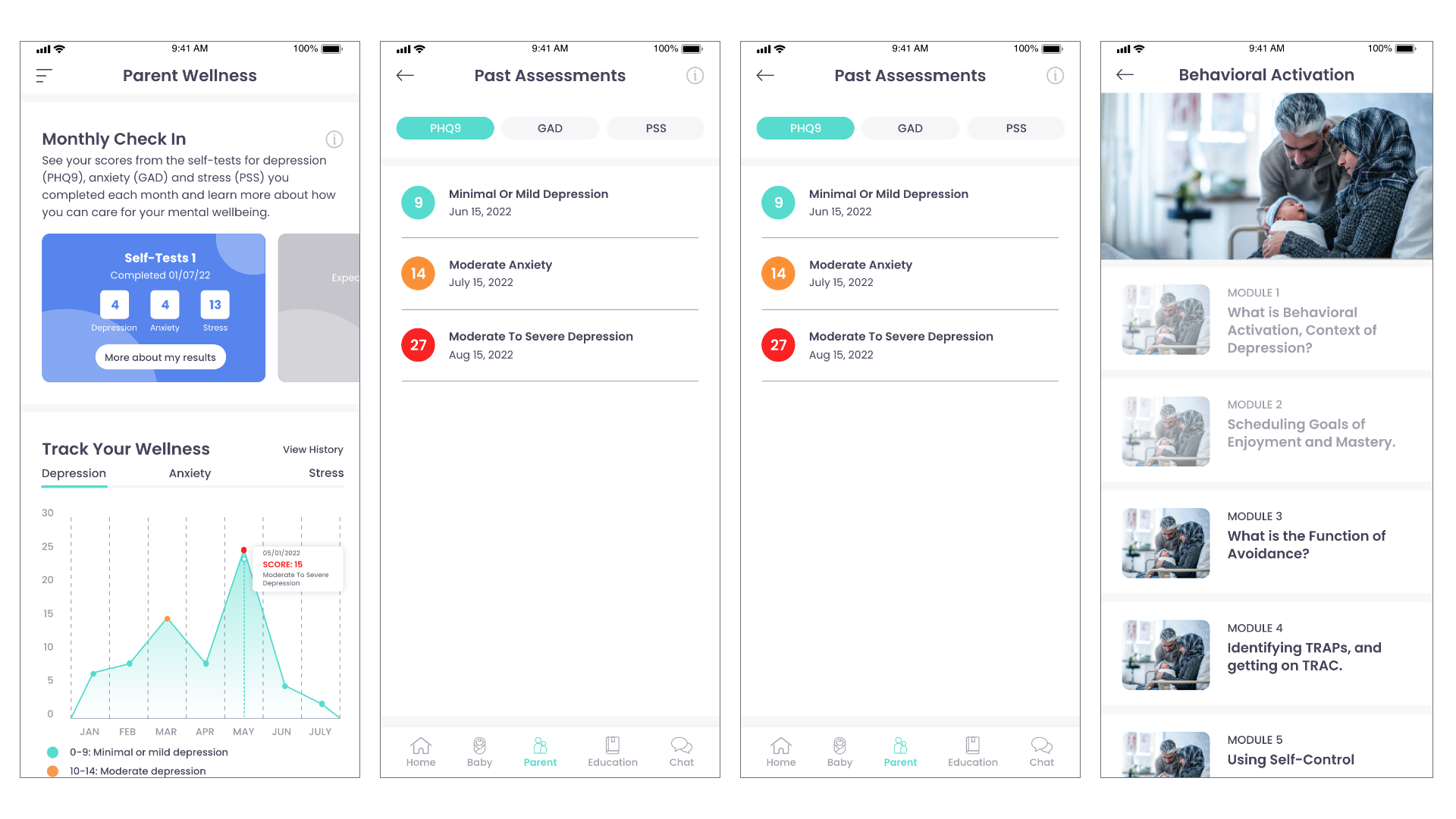 |
| Care manager support | • Live care manager chat for mental health  support needs and baby care needs  • Ongoing monitoring of screening results using the care manager dashboard  • Risk assessment and triage  • Sharing resources with parents surrounding mental health or infant care.  • Helping parents create a personalized care plan  • Helping parents find and connect with a provider covered by their insurance  • Following up with parents to ensure continuity of care and relapse prevention planning | 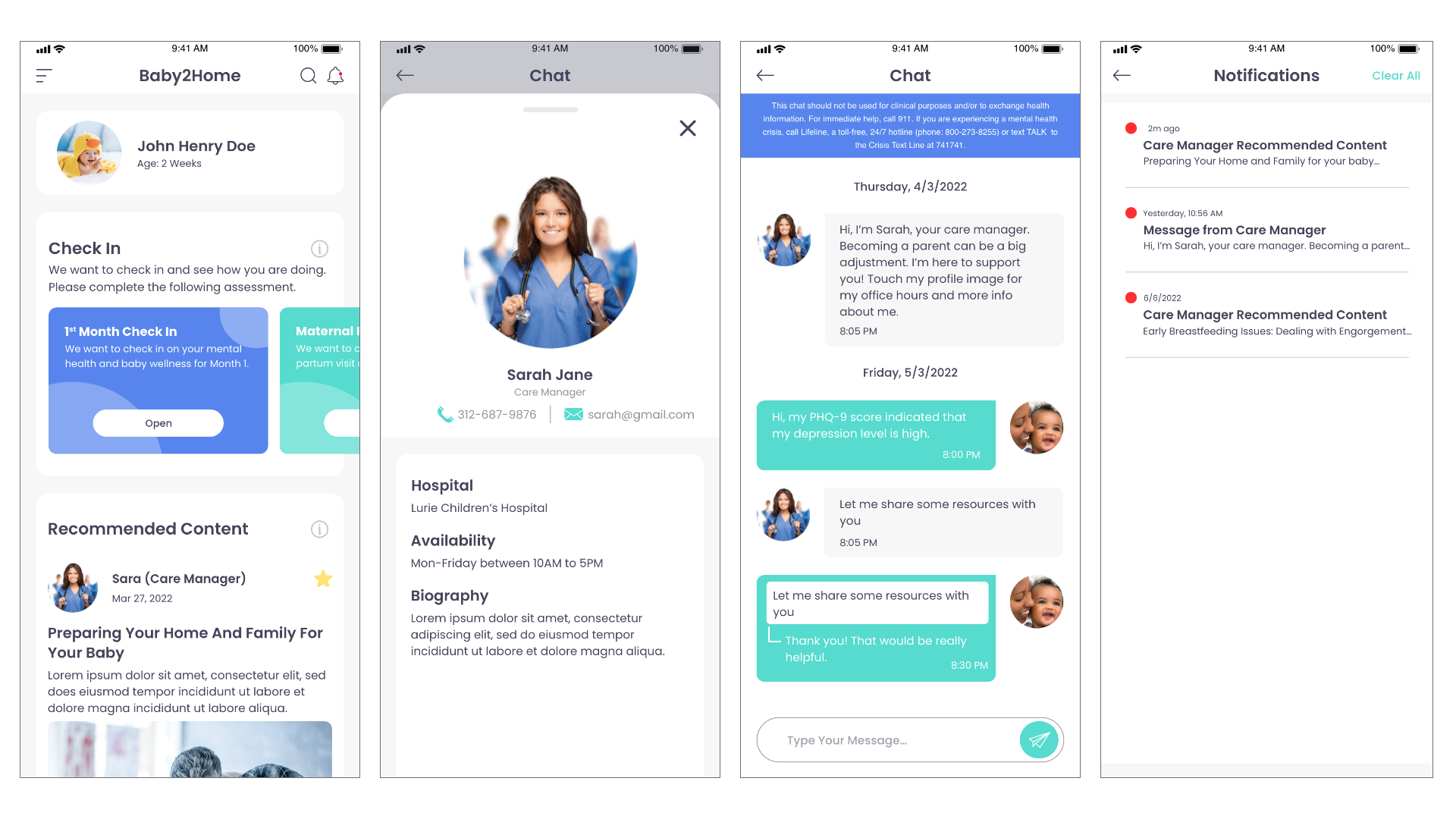  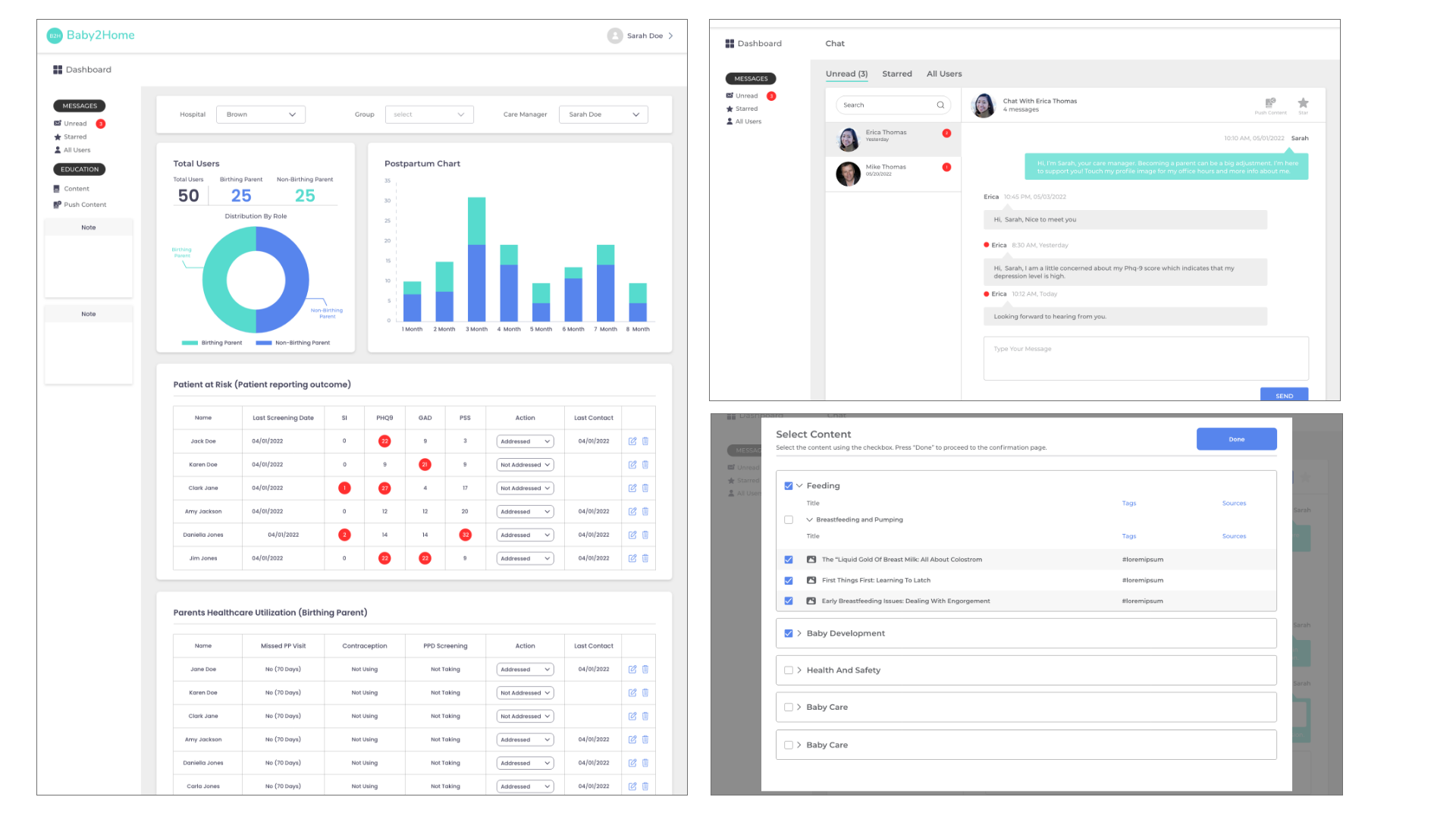 |
