# Supplementary material for: Understanding the Implications of mHealth Technology in Collaborative Care Programs and Its Role in Supporting Postpartum Care: Qualitative Interview Study of the Baby2Home Intervention Using the Parallel Journeys Framework
Source: JMIR Pediatr Parent. 2025 Aug 26;8:e70936. doi: 10.2196/70936 (PMC12421202; doi:10.2196/70936)
Supplement: Multimedia Appendix 2 [file pediatrics_v8i1e70936_app2.docx]

## **Appendix B**

### Summary of the participants and their app usage

| Participant | Role | Race | App Usage | No. Total Session on the app | No. of months postpartum |
| --- | --- | --- | --- | --- | --- |
| P1 | Birthing Parent | European/White | Low Usage | 91 | 8 months |
| P2 | Non-Birthing Parent | European/White | High Usage | 362 | 6 months |
| P3 | Non-Birthing Parent | European/White | High Usage | 178 | 8 months |
| P4 | Birthing Parent | European/White | High Usage | 1778 | 6 months |
| P5 | Birthing Parent | European/White | Low Usage | 150 | 8 months |
| P6 | Birthing Parent | Hispanic /Latina | High Usage | 1518 | 6 months |
| P7 | Birthing Parent | Hispanic /Latina | Low Usage | 69 | 7 months |
| P8 | Birthing Parent | Hispanic /Latina | Low Usage | 36 | 6 months |
| P9 | Birthing Parent | African American | High Usage | 1951 | 8 months |
| P10 | Birthing Parent | European/White | Low Usage | 41 | 8 months |
| P11 | Birthing Parent | European/White | Low Usage | 24 | 4 months |
| P12 | Birthing Parent | European/White | High Usage | 236 | 4 months |
| P13 | Non-Birthing Parent | European/White | Low Usage | 9 | 4 months |
| P14 | Birthing Parent | European/White | High Usage | 3527 | 4 months |
| P15 | Birthing Parent | European/White | Low Usage | 6 | 4 months |
| P16 | Birthing Parent | European/White | High Usage | 2078 | 4 months |
| P17 | Birthing Parent | African American | High Usage | 388 | 4 months |
| P18 | Birthing Parent | European/White | High Usage | 235 | 5 months |
| P19 | Non-Birthing Parent | European/White | High Usage | 480 | 5 months |
| P20 | Birthing Parent | European/White | High Usage | 1297 | 5 months |
