# Supplementary material for: Understanding the Implications of mHealth Technology in Collaborative Care Programs and Its Role in Supporting Postpartum Care: Qualitative Interview Study of the Baby2Home Intervention Using the Parallel Journeys Framework
Source: JMIR Pediatr Parent. 2025 Aug 26;8:e70936. doi: 10.2196/70936 (PMC12421202; doi:10.2196/70936)
Supplement: Multimedia Appendix 3 [file pediatrics_v8i1e70936_app3.docx]

Multimedia appendix 3. Interview guide

**Postpartum Experience:**

- Can you walk us through your experiences with postpartum care and the challenges you faced during this period?
- Did you feel prepared to take care of an infant?
  - If yes, how did you learned what you’ve needed to know about taking care of an infant?
  - If No, what would have helped you feel more prepared to take care of an infant? (class, digital learning, being involved in prenatal care)
- Did you feel prepared to be a parent?
  - If yes, how did you learned what you’ve needed to know about being a parent?
  - If no, what would have helped you feel more prepared to be a parent? (class, digital learning, being involved in prenatal care)

**Understanding Technology Usage:**

- How was your overall experience using B2H and what are some of the factors influencing your app usage?
- How frequently do you use the app on a daily/weekly basis?
  - Has your usage changed since you first downloaded the app?
- How well does the app integrate with your daily routine or activities?
- Are you currently using any other apps that serve a similar purpose to the one we are discussing?
  - If yes, could you please tell me which app(s) and why you use them?
    - How does the app we are discussing compare to other apps you have used in terms of meeting your needs or solving your problems?
    - Are there any specific features or aspects of the other app(s) you are using that you find more appealing or advantageous compared to the app we are discussing?
    - Can you share any insights into why you use multiple apps with similar functions? Are there specific tasks or situations where you prefer one app over the other(s)?
- How was your onboarding experience when you first started using the app?
- When you first downloaded our app, what were your expectations? Did the app meet those expectations?

**Perceived Benefits and Drawbacks of mHealth Intervention**

- What specific features or functionalities of B2H do you find most useful and why?
  - How satisfied are you with the app's functionality and its ability to meet your needs or solve your problems?
- Have you encountered any difficulties or challenges while using the app?
  - If so, can you provide some examples?
  - Have you experienced any technical issues, such as crashes or slow loading times, while using the app?
- Would you recommend B2H to others birthing and non-birthing parents?
  - If yes, what aspects of the app influenced your recommendation?
  - If not, what aspects of the app influenced your recommendation?
- How user-friendly do you find the B2H app? Are there any aspects of the design, navigation, or interface that could be improved to encourage more usage?
- Do you feel that the app meets your needs and expectations regarding perinatal and postpartum care? Why or why not?

**Impact on Healthcare Accessibility and Delivery:**

***Communication:***

- Did the app enhance communication and collaboration between you and your partner? Can you provide specific examples?
  - If yes, what features help facilitate this communication?
  - If no, how do you think communication could be improved within B2H to better cater to your individual needs?
- Did the app enhance communication and collaboration between you and your care manager? Can you provide specific examples?
  - If yes, what features help facilitate this communication?
  - If no, how do you think communication could be improved within B2H to better cater to your individual needs?
- When using the B2H, how well do you feel the communication addressed your specific needs and concerns regarding the following health outcomes:
  - Mental health
  - Preventive health care visits
  - Vaccines
  - Breastfeeding

***Information Seeking:***

- How do you typically seek information related to postpartum health and baby care?
  - Have there been any changes in your information-seeking behaviors since using the Baby2Home?
  - Are there any specific features that have been particularly helpful?
- Have you encountered any challenges in navigating or interpreting the information provided by the app? If so, how do you address these challenges to ensure you receive accurate guidance?
- Are there any aspects of information seeking or the presentation of information in the app that you believe could be improved to better support your postpartum care journey?
- How does the availability of information through the Baby2Home app impact your conversations with healthcare providers?

***Health Service Utilization:***

- Can you tell us a bit about your postpartum journey and any health services you've sought since giving birth?
- In your opinion, how has Baby2Home improved your accessibility to healthcare services during the postpartum period?
  - Can you share any specific instances where the use of the Baby2Home has made accessing healthcare resources or information more convenient for you?
- How valuable are the reminders about relevant content provided by the app? Do you find them helpful in staying informed about your child's needs?
  - Were there any instances where the reminders about relevant content were particularly beneficial or stood out to you? If so, could you describe the situation and how the reminders helped?
  - On the other hand, have you ever felt that the reminders were excessive or unnecessary? If yes, could you explain why you felt that way?
  - How do you think the app could improve its reminders about relevant content to better suit your needs as a parent?
- When it comes to advice about pediatric appointments or changes with your child’s health, how well do you feel the app addresses your concerns or questions?
  - Is there any specific advice or information that you found especially useful in managing appointments for your child's health?
  - Have you ever encountered any challenges in following the advice or suggestions provided by the app regarding appointments? If so, what were they, and how did you handle them?
- For birthing parents- Did you feel like the information regarding postpartum recovery, maternal health and managing your postpartum visit was helpful? If yes, could you explain why?
- For non-birthing parents- Did you find the information regarding the importance of having a primary care doctor and caring for your health to be helpful?
- In terms of breastfeeding, contraception and vaccination information, do you feel that the app has supported you in this area?
  - Has the information been comprehensive and easy to understand?
  - Were there any particular topics or aspects of breastfeeding, contraception or vaccination that you wish the app covered in more detail? If yes, could you provide some examples?

**Engagement with the Educational Feature**

- How has the use of the educational resources provided on B2H impacted your understanding of postpartum health and baby care?
- How familiar were you with postpartum health and baby care concepts before using the Baby2Home mHealth intervention? Can you describe any specific areas where you felt your knowledge improved after using the app?
- Has the app influenced your confidence in managing your postpartum health and baby care responsibilities?
- Did you find the educational content to be up-to-date and relevant to your child's developmental stage or your own postpartum/postnatal stage? Were there any gaps in the information provided?
- How well do you feel B2H caters to your specific information needs?
  - Are there any areas where you think the app could better address your individual health concerns or provide more targeted information?
- Were there any standout features or aspects of the educational resources that you particularly enjoyed or found helpful?
- Did you turn to other platforms to learn more about being home with an infant?
  - What about those resources was more appealing than Baby2Home?
  - What features or resources could Baby2Home add to make it more likely you would use the app instead of the other resource?

**Engagement with the Baby Tracking Feature**

- Can you describe your experience using the baby tracking feature? How has it influenced your daily routine as new parents?
  - What specific aspects of the baby tracking feature do you find most useful or valuable?
  - Have you encountered any challenges or limitations with the baby tracking feature? How do you think these challenges could be addressed or improved?
  - Are there any additional functionalities or improvements you would like to see to make it even more valuable for new parents?
- Can you describe any instances where the baby tracking feature alerted you to a significant change or issue with your baby's health or well-being?
- Has the baby tracking feature provided you with any insights or trends regarding your baby's development or growth milestones?
- Can you share any specific examples of how the baby tracking feature has helped you in communicating with your healthcare provider or pediatrician about your baby's progress and any concerns you may have had?
- Do you feel that the baby tracking feature has improved your overall confidence and knowledge as a new parent? If yes, in what ways?
  - Has it contributed to your overall sense of control and organization as new parents?
  - Have you noticed any reduction in anxiety or uncertainty?
  - Have you noticed any positive changes in your daily routine or organization since using the baby tracking feature?
- How has the baby tracking feature influenced your partner's involvement in baby care and monitoring? Have you both found it beneficial in coordinating tasks and sharing responsibilities?

**Engagement with Treatment and Self-Monitoring Practices (Parent Wellness Feature)**

- Do you feel more engaged and involved in your mental health care due to the integration of Baby2Home? Please explain.
  - In what ways has the app helped or improved your mental health?
- Did you engage with the mental health assessment or the parent wellness feature on the app?
  - - If yes, can you share your overall experience using the parent wellness feature of B2H app?
    - If yes, did you find the app's content, resources, or tools to be relevant and effective in managing your mental health concerns?
    - If not, could you share the reasons why?
- If you needed support with your self-care or mental health, where did you go to learn this information?
  - How come you chose not to use the baby2home app?
  - What features or functionalities would have made it more likely for you to use B2H?
- Were you connected with mental health support or a mental health counselor prior to being enrolled in the Baby2Home study?
- Has the app supported your self-care or self-management practices related to your mental health? (uMars)
  - Did it provide any insights or feedback that have been helpful to you?
  - How did you feel about the monthly mental health surveys with regard to being able to track your scores over time?
  - If no, how would you change the app to make you more likely to use it when you want to support your self care or mental health?
- Have you found the app's content, resources, or tools to be relevant and effective in managing your mental health concerns? Can you provide some examples?
- Have you ever accessed or used the BA education module in the Baby2Home app? If yes, please share your experience. If not, can you explain why you haven't used it?
- Have you ever accessed or used the stress education module in the Baby2Home app? If yes, please share your experience. If not, can you explain why you haven't engaged with it or what factors have discouraged you from using it?
  - In your opinion, how important is stress management for new parents, and do you think the app adequately addresses this need?
  - Do you think the app effectively communicates the benefits of the stress education module? Why or why not?
  - Can you share any challenges or barriers you face in using the stress education module? (e.g., usability issues, time constraints, lack of motivation)
  - Have you sought stress management or parenting advice from sources outside the app? If so, what sources have you used, and why did you choose them over the app's module?
  - Are there any specific aspects of the stress education module that you believe could be improved to make it more appealing or useful to new parents?
  - Can you share any suggestions or ideas for increasing awareness and usage of the stress education module among new parents using the Baby2Home app?
- Were you connected with mental health resources by your Care Manager or another provider?
  - If yes, do you feel like you received the mental health support needed from your Care Manager?
- Have you gotten connected with mental health support since being enrolled in the Baby2Home research study?
  - If yes, how does the app complement or integrate with any other mental health treatments or support you may be receiving (e.g., therapy, medication)?
- What improvements or additional features would you like to see in the app to further enhance its impact on your mental health?

**Engagement with Chat Feature**

- Can you describe your initial expectations and feelings about the chat feature in the Baby2Home app when you first downloaded it?
- How often do you utilize the chat feature in the app to communicate with your care manager?
- Can you describe your overall experience using the chat feature to communicate with your care manager?
  - How has the communication with your care managers been affected by the use of technology? Have you found it easier or more challenging to communicate with them?
  - What specific types of information or support do you typically seek from your care manager through the chat feature?
  - How would you rate the convenience and accessibility of this communication method?
- How satisfied were you with the responsiveness and helpfulness of the care manager's support through the chat feature?
- Has the chat feature allowed you to communicate more openly or comfortably about your concerns or symptoms compared to other forms of communication? Why or why not?
- Do you feel that the chat feature adequately addresses your communication needs with your care manager, or are there additional features or improvements you would like to see in the future?
